# Relationship between smoking and postoperative complications of cervical spine surgery: a systematic review and meta-analysis

**DOI:** 10.1038/s41598-022-13198-x

**Published:** 2022-06-02

**Authors:** Li-ming Zheng, Zhi-wen Zhang, Wei Wang, Yang Li, Feng Wen

**Affiliations:** 1 College of Acupuncture and Orthopedics, Hubei University of Chinese Medicine, Wuhan, 430061 China; 2 Department of Orthopedics, Hubei Provincial Hospital of Traditional Chinese Medicine, Affiliated to Hubei University of Chinese Medicine, 4 Garden Hill, Wuchang, Wuhan, 430061 Hubei China; 3 Affiliated Hospital of Hubei University of Chinese Medicine, Wuhan, China; 4 Hubei Provincial Academy of Traditional Chinese Medicine, Wuhan, 430070 China

**Keywords:** Diseases, Medical research

## Abstract

To determine whether smoking has adverse effects on postoperative complications following spine cervical surgery (PROSPERO 2021: CRD42021269648). We searched PubMed, Embase, Cochrane Library, and Web of Science through 13 July 2021 for cohort and case–control studies that investigated the effect of smoking on postoperative complications after cervical spine surgery. Two researchers independently screened the studies and extracted data according to the selection criteria. The meta-analysis included 43 studies, including 27 case–control studies and 16 cohort studies, with 10,020 patients. Pooled estimates showed that smoking was associated with overall postoperative complications (effect estimate [ES] = 1.99, 95% confidence interval [CI]: 1.62–2.44, *p* < 0.0001), respiratory complications (ES = 2.70, 95% CI: 1.62–4.49, *p* < 0.0001), reoperation (ES = 2.06, 95% CI: 1.50–2.81, *p* < 0.0001), dysphagia (ES = 1.49, 95% CI: 1.06–2.10, *p* = 0.022), wound infection (ES = 3.21, 95% CI: 1.62–6.36, *p* = 0.001), and axial neck pain (ES = 1.98, 95% CI: 1.25–3.12, *p* = 0.003). There were no significant differences between the smoking and nonsmoking groups in terms of fusion (ES = 0.97, 95% CI: 0.94–1.00, *p* = 0.0097), operation time (weighted mean difference [WMD] = 0.08, 95% CI: −5.54 to 5.71, *p* = 0.977), estimated blood loss (WMD = −5.31, 95% CI: −148.83 to 139.22, *p* = 0.943), length of hospital stay (WMD = 1.01, 95% CI: −2.17 to 4.20, *p* = 0.534), Visual Analog Scale-neck pain score (WMD = −0.19, 95% CI: −1.19 to 0.81, *p* = 0.707), Visual Analog Scale-arm pain score (WMD = −0.50, 95% CI: −1.53 to 0.53, *p* = 0.343), Neck Disability Index score (WMD = 11.46, 95% CI: −3.83 to 26.76, *p* = 0.142), or Japanese Orthopedic Association Scores (WMD = −1.75, 95% CI: −5.27 to 1.78, *p* = 0.332). Compared with nonsmokers, smokers seem to be more significantly associated with overall complications, respiratory complications, reoperation, longer hospital stay, dysphagia, wound infection and axial neck pain after cervical spine surgery. It is essential to provide timely smoking cessation advice and explanation to patients before elective cervical spine surgery.

## Introduction

Cigarette smoking is a significant public health concern worldwide. Approximately 20% of adults in the US currently smoke cigarettes, which are responsible for up to 20% of all deaths each year^1^. In some cervical surgeries, more than half of the patients are smokers^2–4^. Smoking is highly detrimental to health and is associated with cancer, respiratory disease, and cardiovascular disease^5^. A growing body of evidence shows that smoking is a significant risk factor for adverse surgical outcomes after spine surgery^5–8^.

The relationship between smoking and outcomes of cervical surgery has not been well evaluated. Some studies suggest that smoking may be associated with poorer outcomes after cervical surgery, including lower fusion rates^9,10^. Smoking has been independently linked to higher volumes of blood loss^11^, longer lengths of stay^2,11^, and higher reoperation rates^12,13^. There is also an increased risk of perioperative complications, including dysphagia, airway obstruction, nerve palsy, reintubation, axial neck pain, wound infection, deep venous thrombosis, pneumonia, and pseudarthrosis^7,11,12,14–17^. Pain control and functional outcomes have also been shown to be less favorable in smoking patients^18,19^.

Nevertheless, some studies disputed these findings and suggested no relationship between smoking and adverse surgical outcomes after cervical surgery^18,20,21^. Some researchers even found that the incidence of complications in smokers was lower than that in nonsmokers after posterior cervical fusion^22^. We performed the present study to resolve these discrepancies. To the best of our knowledge, there have been no previous systematic reviews and meta-analyses that assess the association between smoking and outcomes of cervical spine surgery.

## Materials and methods

### Literature search strategy

This meta-analysis was performed in accordance with the Meta-analysis of Observational Studies in Epidemiology (MOOSE) statement^23^. The PubMed, Embase, Cochrane Library, and Web of Science electronic databases were searched from inception to 13 July 2021 using the MeSH terms “smoking,” “cervical vertebrae,” “surgical procedures, operative,” and their corresponding free terms. The search was restricted to human subjects. In addition, we also reviewed the list of references for retrieved papers and recent reviews.

### Inclusion and exclusion criteria

The inclusion criteria were as follows: (1) The study design was cohort studies, case–control studies, or controlled or comparative studies; (2) the study population consisted of smokers and nonsmokers who underwent cervical spine surgery; and (3) the study compared outcomes, including operating time, pain score, functional score, reoperation, length of hospital stay, estimated blood loss, fusion, and postoperative complications. The exclusion criteria were as follows: (1) reviews, letters, case reports, systematic reviews, animal studies, noncomparative studies, and studies that were unrelated to our topics; (2) the study did not involve any of the outcomes listed in the inclusion criteria; and (3) duplicated publications from the same hospital or research center. For accepted articles that covered the same population or subpopulation, the most informative articles or complete studies were used to prevent duplication of information.

### Data extraction

Data extraction was conducted according to the Preferred Reporting Items for Systematic Reviews and Meta-Analyses (PRISMA) statement, and the selection of articles and the extraction of data were carried out independently by two reviewers and examined by other authors. Any disagreements were resolved by consensus or discussion with a third reviewer. The following information was extracted from the studies: (1) the general study information (name of the first author, publishing date, country, study design, sample size, age, sex, surgical procedure, follow-up time, and definition of smoking); (2) perioperative parameters, including operative time, estimated blood loss, and length of hospital stay; (3) clinical outcomes, including visual analog scale (VAS) scores of neck pain and arm pain, Neck Disability Index (NDI) score, and Japanese Orthopedic Association Scores (JOA); (4) complications, fusion and reoperation; the complications were defined as primary outcomes in this study, including dysphagia, airway obstruction, nerve palsy, reintubation, axial neck pain, wound infection, deep venous thrombosis, pneumonia, and pseudarthrosis. For continuous outcomes, we extracted the mean and standard deviation, and participant numbers were also extracted. For dichotomous outcomes, we extracted the total numbers and the numbers of events of both groups. The data in other forms was recalculated when possible to enable pooled analysis.

### Methodological quality

Reviewers applied the Newcastle–Ottawa Scale (NOS) to evaluate the methodological quality of the included studies^24^. The NOS is a scoring checklist for solving design and implementation issues of a cohort or case–control study, which consisted of participant selection, comparability of cases and controls, exposure, and outcomes. If the study was awarded six or more stars, it was considered a high-quality study and was analyzed.

### Statistical analysis

We used STATA version 12.0 (StataCorp, College Station, TX) to generate forest plots to determine whether there was a statistical association between the case and control groups and to assess heterogeneity of the included studies. Dichotomous outcomes were expressed as effect estimates (ESs) with 95% confidence intervals (CIs); among them, the results of case–control studies are expressed as odds ratios (ORs), and the results of cohort studies are expressed as relative risks (RRs); continuous outcomes are expressed as the weighted mean differences (WMDs). Heterogeneity was quantified using the chi-square based Cochran’s Q statistic^25^ and the I^2^ statistic, which yields results ranged from 0 to 100% (I^2^ = 0–25%, no heterogeneity; I^2^ = 25–50%, moderate heterogeneity; I^2^ = 50–75%, large heterogeneity; and I^2^ = 75–100%, extreme heterogeneity)^26^. In cases of substantial heterogeneity, the random-effects model was applied. Otherwise, the fixed-effects model was used. When heterogeneity was present, a ‘leave-one-out sensitivity analysis was performed by iteratively removing one study at a time to confirm the source of the heterogeneity. Analysis was then performed without the study to determine if heterogeneity was still present and if so, random-effects modeling was used. Publication bias was assessed using visual inspection of the funnel plot with the Begg^27^ and Egger tests^28^. All statistical tests were two-sided, and *p*-values of < 0.05 were considered statistically significant.

## Results

### Identification of eligible studies

**Figure 1:**
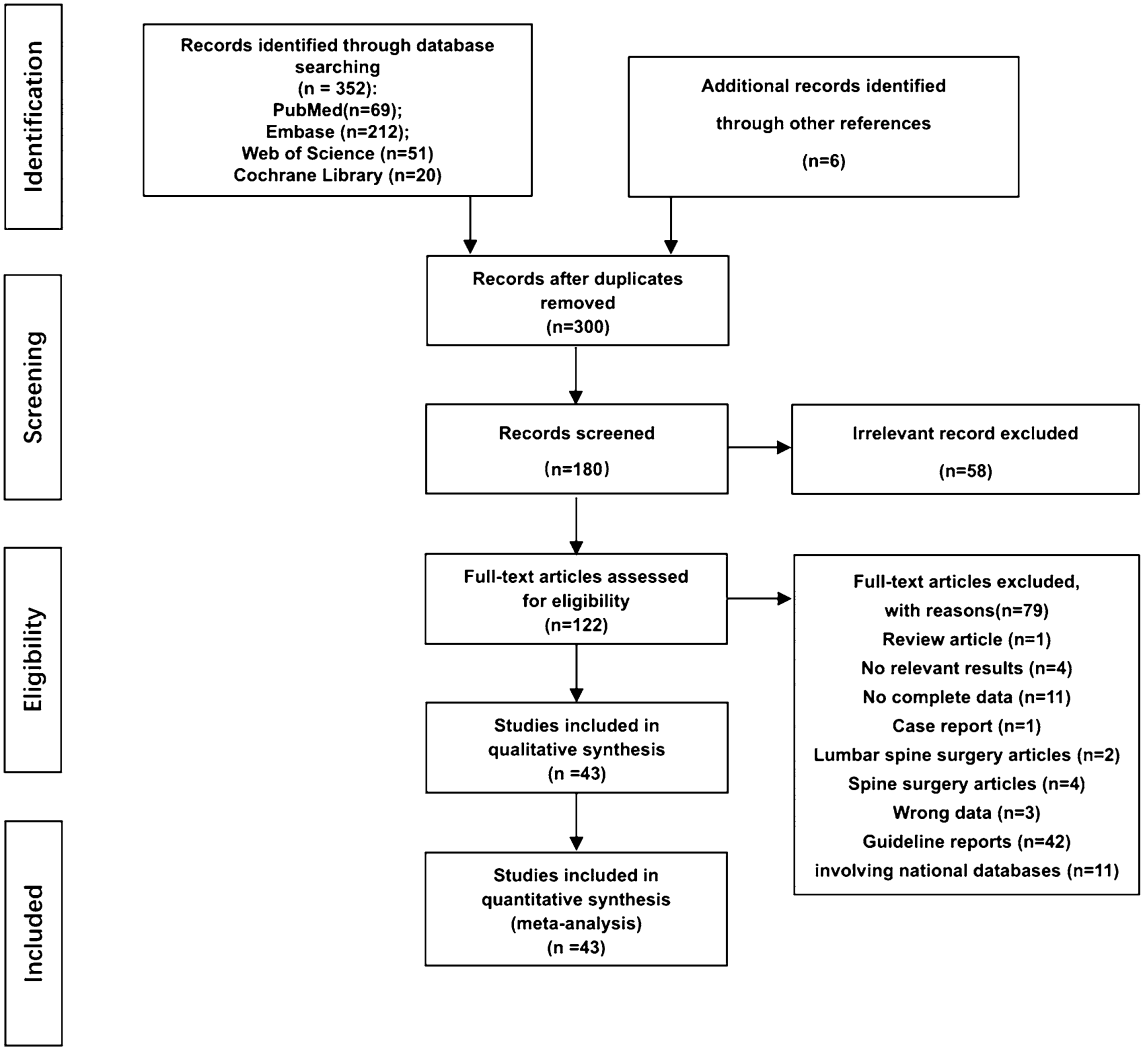
Flow diagram of study selection.

A flowchart of the search and study selection process is shown in Fig. 1. The electronic search identified a total of 352 citations (69 from PubMed, 212 from EMBASE, 20 from the Cochrane Library, and 51 from the Web of Science). After screening titles and abstracts and removal of duplicates, 122 were considered of interest; the full text of these 122 studies was retrieved for detailed evaluation; 79 studies were excluded, and 43 studies were ultimately included in the meta-analysis^2–4,7,9–16,18–21,29–55^.

### Characteristics of included studies

The characteristics of the studies are summarized in Table 1. The 43 independent observational studies included in this meta-analysis were published from 1995 to 2021. These forty-three studies included 10,020 patients, including 3,107 smokers and 6,913 nonsmokers. Twenty-seven studies were conducted in the United States and seven were conducted in China. The other nine were conducted in India, Japan, the Czech Republic, Italy, Korea, Singapore, and Taiwan. Of these, 16 were cohort studies, and 27 were case–controls. All raw data are available in the Supplementary Tables S1 and S2.

**Table 1:** Baseline characteristics of included studies.

Series (year)	Country	Design	Number of patients		Age (mean ± SD, year)	Gender, number		Surgery	Definition of smoking	Follow-up (mean ± SD)	NOS score	Pool of outcomes of included studies
			Smoker	Nonsmoker		Male	Female					
Agrillo et al.^30^	Italy	Case–control	19	26	49.7 (28–77)	26	19	ACDF	Smoking history	6 months	6	Fusion
An et al.^31^	USA	Cohort	34	43	47.1	NR	Anterior cervical fusion	NR	12–13 months	6	Fusion	
Badiee et al.^12^	USA	Case–control	27	232	63.2 ± 10.8	129	130	Posterior cervical decompression and fusion	NR	90 days	8	Wound infection, neurological deficit, seroma, reoperation
Bergin et al.^32^	USA	Case–control	48	278	53.8	149	177	ACDF	NR	27.6 ± 19.0 months	8	Fusion
Bose et al.^20^	USA	Cohort	46	60	50.12 ± 11.72 (27–80)	47	59	ACDF	NR	> 12 months	8	Deltoid weakness, airway obstruction, dysphagia, recurrent laryngeal nerve palsy, reoperation
Cerier et al.^19^	USA	Cohort	23	38	50.4	32	29	ACDF	Smoking within 6 months before surgery	6 months	7	NDI, fusion
Chen et al.^33^	China	Case–control	68	189	NR	138	119	Single-level anterior cervical fusion	Smoking history	6–24 months	8	Dysphagia
Dube et al.^34^	India	Case–control	44	163	3 mo–86 y	160	47	Cervical Spine Surgery	NR	NR	7	Pulmonary complications (dyspnea, pneumonia, tracheobronchitis, arterial desaturation, reintubation, atelectasis, pleural effusion, pneumothorax, acute respiratory distress syndrome)
Eubanks et al.^35^	USA	Cohort	41	117	61	93	65	Posterior cervical fusion	NR	14.5 (3–72) months	8	C5 palsy, wound infection
Goldberg et al.^36^	USA	Cohort	30	50	44.6	43	37	ACDF	NR	4 (2–7) years	6	Fusion
Groff et al.^37^	USA	Case–control	55	89	49	119	25	Partial corpectomy and fusion	Smoking within 3 months before surgery	34 (> 24) months	6	Fusion
Hilibrand et al.^9^	USA	Cohort	55	135	NR	NR	ACDF	NR	68 (24–183) months	7	Fusion	
Huang et al.^16^	China	Case–control	51	130	52.15 ± 9.32	104	77	ACDF	NR	18 (12–24) months	8	Dysphagia
Kang et al.^38^	Korea	Case–control	41	31	47.1 ± 7.8	50	22	ACDF	Smoking history	1 year	7	Dysphagia
Kimura et al.^39^	Japan	Case–control	39	117	64	108	48	Laminoplasty	Current smoking	2 years	7	Axial neck pain
Klement et al.^40^	USA	Case–control	2	27	63	8	21	Cervical laminectomy and fusion	NR	26.9 months	8	C5 palsy
Lau et al.^11^	USA	Cohort	62	70	NR	77	55	Anterior cervical corpectomy and fusion	Smoking history	1 year	8	Wound infection, seroma, Implant failure, CSF leakage, DVT, urinary tract infection, pneumonia, epidural hematoma, bacteremia, airway obstruction, reintubation, myocardial infarction, pulmonary edema, hepatic encephalopathy, acute renal failure, hardware failure, sepsis, meningitis, pericardial effusion, pleural effusion, reoperation, EBL, length of stay
Lee et al.^41^	USA	Case–control	403	955	51 (20–91)	729	629	Cervical Spine surgery	NR	12–168 months	7	Reoperation
Lee et al.^42^	Korea	Case–control	333	705	50 (22–89)	514	524	Anterior cervical surgery	NR	50 (12–168) months	7	Adjacent segment pathology
Liang et al.^43^	China	Case–control	59	158	55.4	109	108	Anterior cervical corpectomy and fusion	Smoking history	NR	7	Increased surgical drain output
Liu et al.^44^	China	Case–control	39	49	60.4	45	43	ACDF	NR	1 year	8	Axial neck pain
Luszczyket al. ^45^	USA	Cohort	156	417	NR	NR	ACDF	Current smoking	> 24 months	6	Fusion	
Mangan et al.^46^	USA	Cohort	63	87	53	123	141	ACDF	Smoking history	19.8 (9–20.6) months	8	Fusion, reoperation
Martin et al.^10^	USA	Cohort	75	214	NR	162	127	ACDF	Smoking history	33 (24–51) months	8	Fusion
Nakashima et al.^47^	Japan	Case–control	55	109	44.9 (14–90)	142	22	Posterior cervical surgery	Smoking history	59.9 months	7	Tracheostomy
Pahys et al.^14^	USA	Case-contorl	126	357	53.7	268	215	Posterior cervical spine surgery	Smoking history	> 3 months	8	Wound infection
Patel et al.^48^	USA	Cohort	25	167	48.7	115	77	ACDF	NR	6 months	8	VAS neck pain, VAS arm pain, NDI, operative time, EBL, length of stay
Plano et al.^49^	USA	Case–control	128	175	57.7 ± 12.6 (27–86)	200	103	Cervical Spine surgery	NR	75.35 ± 27.1 (16–126) months	7	Reoperation
Reinard et al.^2^	USA	Case–control	47	30	55.1 ± 12.88 (20–86)	50	27	Combined anterior–posterior cervical spinal fusions	Smoking history	NR	8	Dysphagia, EBL, length of stay
Ren et al.^50^	China	Case–control	106	189	59.7	139	156	ACDF	NR	6 months	8	Fusion
Riederman et al.^7^	USA	Case–control	36	164	52.4 (28–87)	112	88	ACDF	Smoking history	NR	7	Dysphagia
Sagi et al.^52^	USA	Case–control	127	184	47	169	142	Anterior cervical surgery	NR	NR	7	Airway complications (reintubation, airway obstruction)
Schnee et al.^29^	USA	Case–control	66	78	48.1 (27–82)	71	73	Anterior cervical fusion	NR	8.1 (2.7–34.2) months	6	Wound infection
Siemionow et al.^7^	USA	Case–control	16	19	60 (37–82)	21	14	Combined anterior–posterior cervical spinal fusions	NR	> 12 months	6	Wound infection, reintubation
Suchomel et al.^53^	Czech Republic	Cohort	48	31	47.8 (37–73)	49	30	ACDF	Smoking history	39.4 (24–48) months	7	Fusion
Tu et al.^18^	Taiwan, China	Cohort	20	89	47.5	56	53	Cervical disc arthroplasty	Smoking within 6 months before surgery	42.3 (> 24) months	8	Neurological deficit, C5 palsy, hoarseness, dysphagia, wound infection, CSF leakage, VAS neck pain, VAS arm pain, NDI, JOA
Vasquez et al.^21^	USA	Cohort	123	350	18–70	267	206	Cervical Spine Surgery	Current smoking	12 months	8	VAS neck pain, VAS arm pain, NDI, operative time, length of stay
Wang et al.^54^	USA	Cohort	12	68	43.3 (19–70)	33	74	ACDF	NR	2.3 (1–6) years	6	Fusion
Wang et al.^55^	USA	Case–control	6	52	47.6 (25–90)	26	34	ACDF	NR	2.7 (1–6) years	6	Fusion
Wang et al.^3^	China	Case–control	46	22	67.6	29	39	Anterior cervical surgery	NR	1 year	8	Dysphagia
Wen-Shen et al.^13^	Singapore	Cohort	20	117	45.8	66	71	Cervical artificial disc replacement	Current smoking	74 (> 24) years	8	Reoperation
Woodroffe et al.^4^	USA	Case–control	219	151	57.8	231	139	Posterior cervical fusion	Smoking history	NR	8	Reoperation
Zhang et al.^15^	China	Case–control	68	181	60.5 ± 7.6	120	129	Laminoplasty	NR	12–108 months	8	Axial neck pain

*NR* not reported, *NOS* Newcastle–Ottawa Scale, *ACDF* anterior cervical discectomy and fusion, *SD* standard deviation, *NDI* neck disability index, *VAS* visual analog scale, *JOA* Japanese Orthopaedic Association Scores for Assessment of Cervical Myelopathy, *EBL* estimated blood loss, *CSF* cerebrospinal fluid, *DVT* deep venous thrombosis.

### Quality of included studies

Because all the included studies were cohort studies or case–control studies, the quality of each study was evaluated using the NOS (maximum of nine stars). Case–control studies were divided into three categories: selection, comparability, and exposure, and cohort studies were divided into three categories: selection, comparability, and outcomes. According to the NOS scale, all included studies were considered to be of high-quality: 12 were awarded eight stars, 10 were awarded seven stars, and 5 were awarded six stars in case–control studies (Table 2). Nine were awarded eight stars, 3 were awarded seven stars, and 4 were awarded six stars in cohort studies (Table 3).

**Table 2:** Quality assessment of case–control studies according to Newcastle–Ottawa scale.

Study	Selection	Comparability	Exposure	Total
Agrillo et al.^30^	****		**	6
Badiee et al.^12^	****	**	**	8
Bergin et al.^32^	****	**	**	8
Chen et al.^33^	****	**	**	8
Dube et al.^34^	****	**	*	7
Groff et al.^37^	****	*	*	6
Huang et al.^16^	****	**	**	8
Kang et al.^38^	****	**	*	7
Kimura et al.^39^	****	**	*	7
Klement et al.^40^	****	**	**	8
Lee et al.^41^	****	**	*	7
Lee et al.^42^	****	**	*	7
Liang et al.^43^	****	**	*	7
Liu et al.^44^	****	**	**	8
Nakashima et al.^47^	****	**	*	7
Pahys et al.^14^	****	**	**	8
Plano et al.^49^	****	*	**	7
Reinard et al.^2^	****	**	**	8
Ren et al.^50^	****	**	**	8
Riederman et al.^7^	****	*	**	7
Sagi et al.^52^	****	*	**	7
Schnee et al.^29^	****		**	6
Siemionow et al.^7^	****		**	6
Wang et al.^55^	****		**	6
Wang et al.^3^	****	**	**	8
Woodroffe et al.^4^	****	**	**	8
Zhang et al.^15^	****	**	**	8

**Table 3:** Quality Assessment of Cohort Studies According to Newcastle–Ottawa Scale.

Study	Selection	Comparability	Outcome	Total
An et al.^31^	***		***	6
Bose et al.^20^	***	**	***	8
Cerier et al.^19^	***	*	***	7
Eubanks et al.^35^	***	**	***	8
Goldberg et al.^36^	***		***	6
Hilibrand et al.^9^	***	*	***	7
Lau et al.^11^	****	*	***	8
Luszczyk et al.^45^	***		***	6
Mangan et al.^46^	***	**	***	8
Martin et al. ^10^	****	*	***	8
Patel et al. ^48^	***	**	***	8
Suchomel et al.^53^	****		***	7
Tu et al.^18^	***	**	***	8
Vasquez et al.^21^	***	**	***	8
Wang et al.^54^	***		***	6
Wen-Shen et al.^13^	***	**	***	8

### Meta-analysis

#### Overall complications

**Figure 2:**
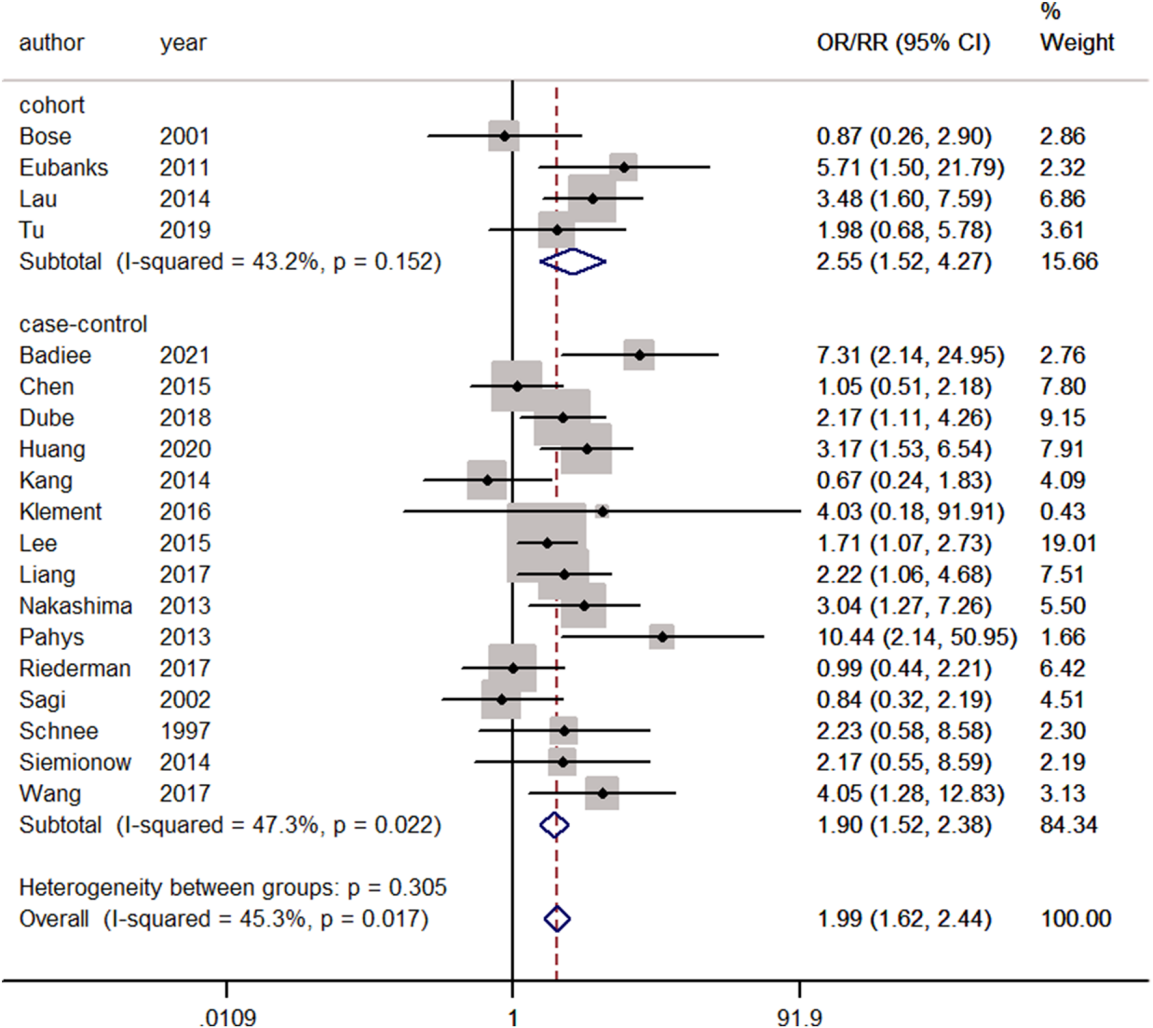
Forest plot showing the effect of smoking on overall complications. OR, odd rate; RR, risk rate; CI, confidence interval.

The primary outcomes in our meta-analysis were complications, including dysphagia, airway obstruction, nerve palsy, reintubation, axial neck pain, wound infection, deep venous thrombosis, pneumonia, deltoid weakness, tracheobronchitis, and pseudarthrosis. At least one postoperative complication was reported in 20 studies^2,3,11,12,14,16,18,20,21,29,33–35,38,40,42,43,47,51,52^. Significant heterogeneity was observed, and the random-effects model was used (I^2^ = 50.8%, *p* = 0.005). The meta-analysis revealed that smokers were significantly associated with postoperative complications compared with nonsmokers (ES = 1.96, 95% CI: 1.45–2.66, *p* < 0.001). Because of the heterogeneity (I^2^ = 50.8%), a sensitivity analysis was performed. The study of Reinard et al.^2^ excluded patients with a recombinant human bone morphogenetic protein associated with dysphagia after cervical surgery^56^. It may affect the incidence of postoperative dysphagia. Excluding this paper reduced I^2^ to 45.3% (Fig. 2). Reanalysis using a fixed-effects model revealed that, compared with nonsmokers, smokers were significantly associated with postoperative complications (ES = 1.99, 95% CI: 1.62–2.44, *p* < 0.0001).

#### Respiratory complications

**Figure 3:**
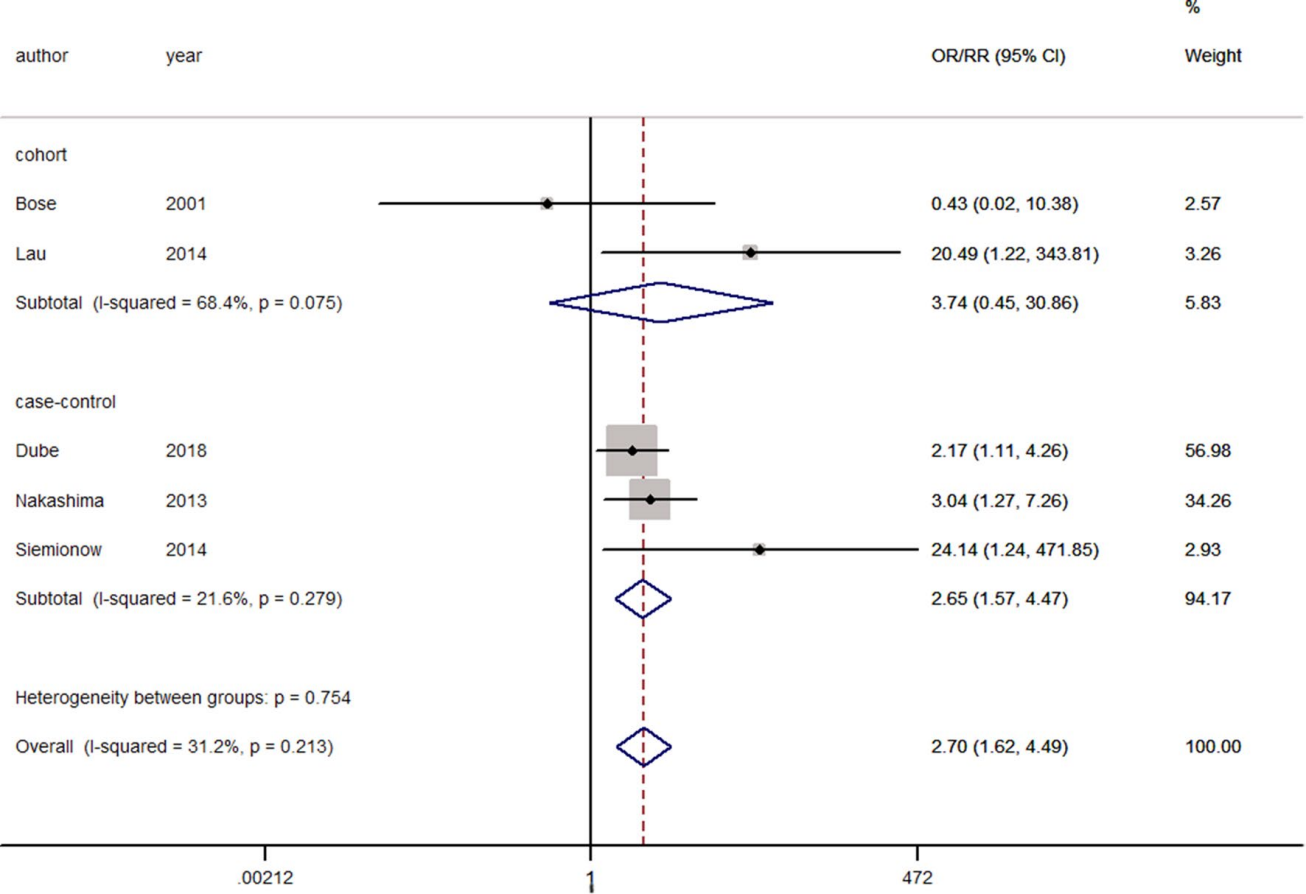
Forest plot showing the effect of smoking on respiratory complications. OR, odd rate; RR, risk rate; CI, confidence interval.

Six studies reported postoperative respiratory complications, including dyspnea, reintubation, airway obstruction, pneumonia, and tracheotomy^7,11,20,34,47,52^. There was significant heterogeneity (I^2^ = 51.4%, *p* = 0.068); therefore, the random-effects model was used. Pooling of the results demonstrated that smokers were more associated with respiratory complications than nonsmokers (ES = 2.30, 95% CI: 1.05–5.05, *p* = 0.038). After performing sensitivity analysis and removing the study by Sagi et al.^52^ a higher proportion of patients who had exposure of C4 or above compared with other studies, the heterogeneity was reduced to 31.2% (Fig. 3). Fixed-effects modeling showed that smokers were significantly more associated with respiratory complications than nonsmokers (ES = 2.70, 95% CI: 1.62–4.49, *p* < 0.0001).

#### Reoperation

**Figure 4:**
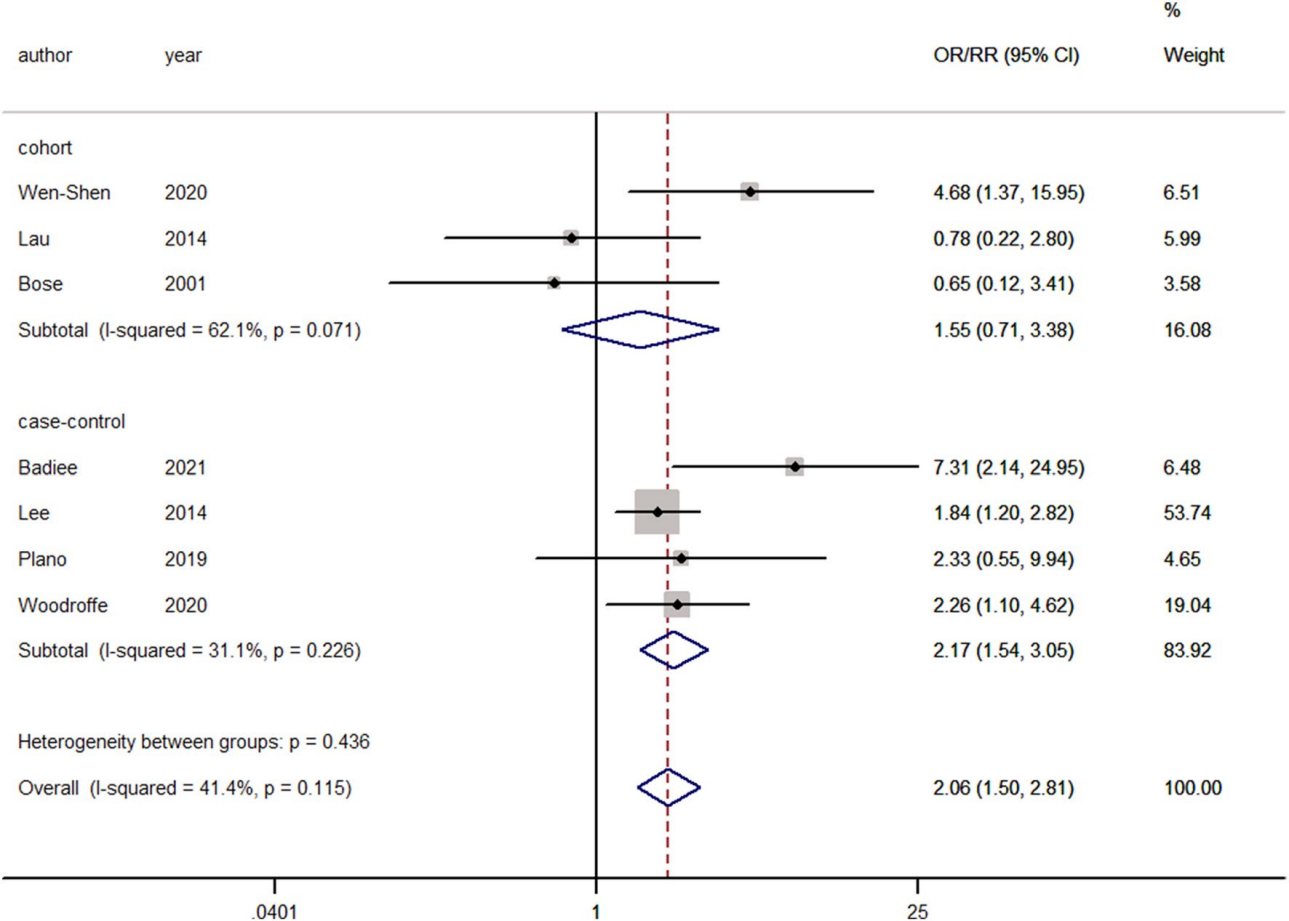
Forest plot showing the effect of smoking on reoperation. OR, odd rate; RR, risk rate; CI, confidence interval.

The number of patients who underwent reoperation was provided in eight studies^4,11–13,20,41,46,49^. Significant heterogeneity was observed, and a random-effects model was used (I^2^ = 57.7%, *p* = 0.020). Pooling of the results demonstrated that smokers were more associated with reoperation than nonsmokers (ES = 1.80, 95% CI: 1.06–3.06, *p* = 0.0029). When performing statistical analysis of Mangan et al.^46^, we defined the sum of current and former smokers as the total number of smokers. We then removed Mangan et al., performed a sensitivity analysis, and found that heterogeneity was reduced to 41.4% (Fig. 4). Reanalysis using a fixed-effects model revealed that smokers were significantly more associated with reoperation after cervical spine surgery than nonsmokers (ES = 2.06, 95% CI: 1.50–2.81, *p* < 0.001).

#### Fusion

**Figure 5:**
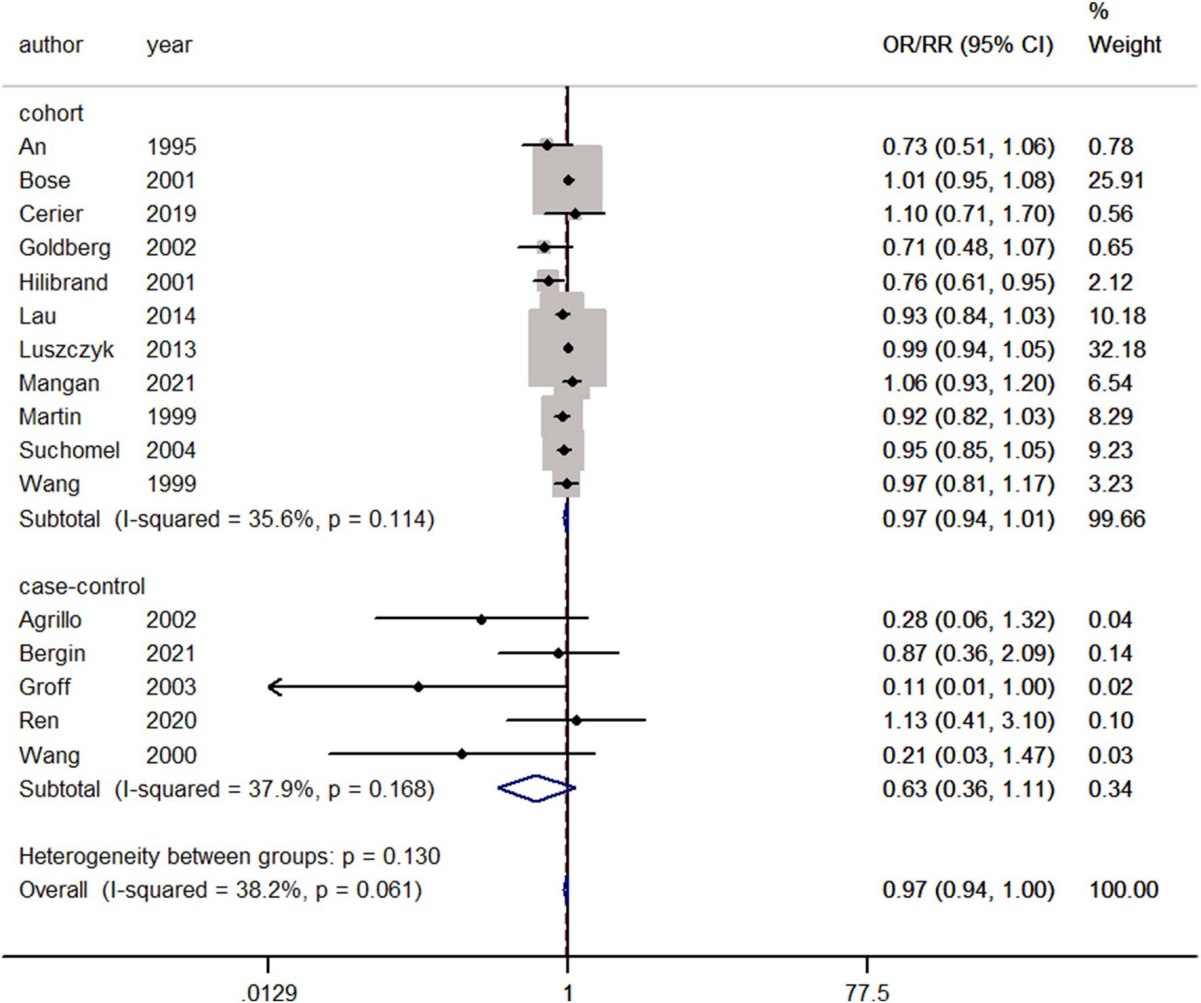
Forest plot showing the effect of smoking on fusion. OR, odd rate; RR, risk rate; CI, confidence interval.

Sixteen studies performed cervical fusion surgery and reported fusion^9–11,19,20,30–32,36,37,45,46,50,53–55^. No significant heterogeneity was observed, and a fixed-effects model was used (I^2^ = 38.2%, *p* = 0.061). Pooling of the results demonstrated that after cervical fusion surgery, there was no significant difference in fusion between the smoking group and the nonsmoking group (ES = 0.97, 95% CI: 0.94–1.00, *p* = 0.097; Fig. 5).

#### Dysphagia

**Figure 6:**
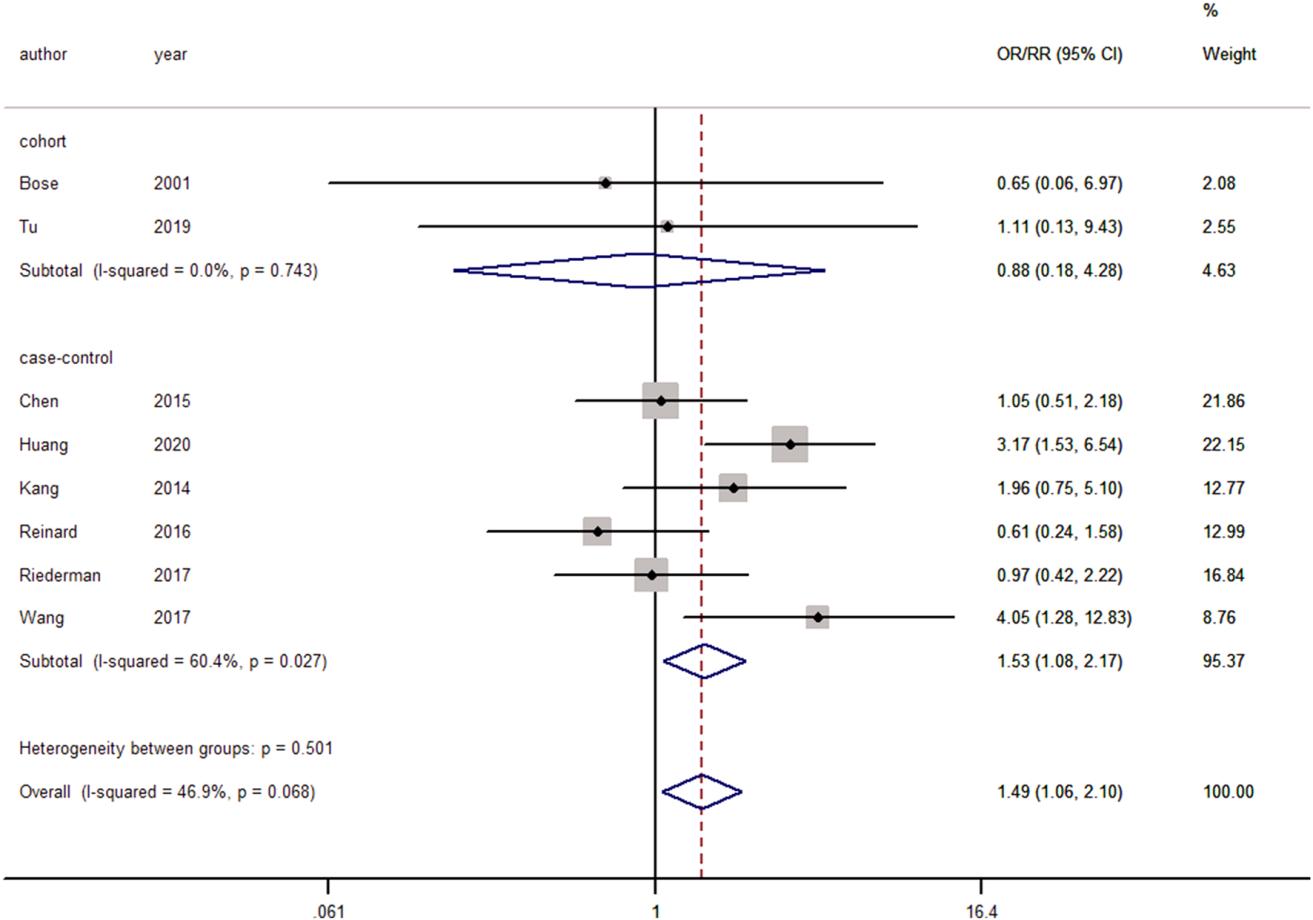
Forest plot showing the effect of smoking on dysphagia. OR, odd rate; RR, risk rate; CI, confidence interval.

Eight studies reported dysphagia after cervical spine surgery^2,3,16,18,20,33,51^. No significant heterogeneity was observed, and a fixed-effects model was used (I^2^ = 46.9%, *p* = 0.0068). Pooling of the results demonstrated that smokers were more associated with postoperative dysphagia than nonsmokers (ES = 1.49, 95% CI: 1.06–2.10, *p* = 0.022; Fig. 6).

#### Wound infection

**Figure 7:**
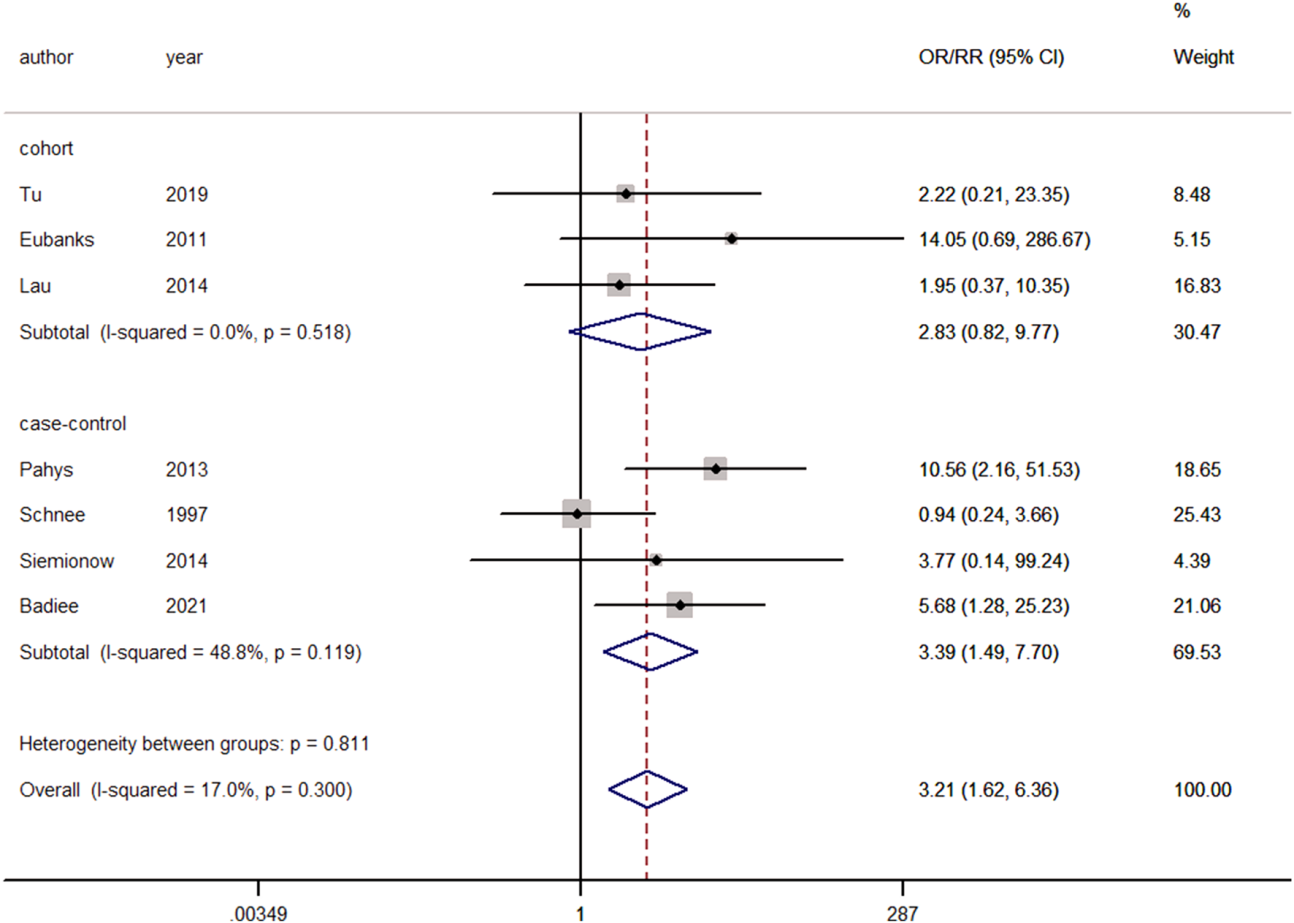
Forest plot showing the effect of smoking on wound infection. OR, odd rate; RR, risk rate; CI, confidence interval.

Seven studies reported postoperative wound infection^7,11,12,14,18,29,35^. No significant heterogeneity was observed, and a fixed-effects model was used (I^2^ = 17.0%, *p* = 0.300). Pooling of the results demonstrated that smokers were significantly more associated with postoperative wound infection than nonsmokers (ES = 3.21, 95% CI: 1.62–6.36, *p* = 0.001; Fig. 7).

#### Axial neck pain

**Figure 8:**
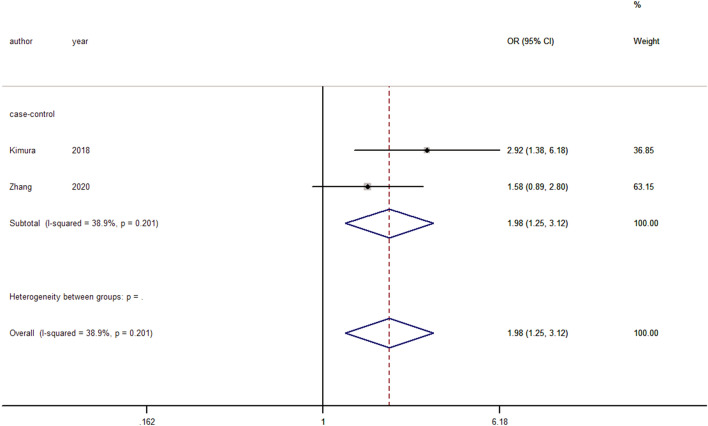
Forest plot showing the effect of smoking on axial neck pain. OR, odd rate; RR, risk rate; CI, confidence interval.

Three studies reported postoperative axial neck pain^15,39,44^. Significant heterogeneity was observed, and a random-effects model was used (I^2^ = 63.7%, *p* = 0.064). Pooling of the results shows that compared with nonsmokers, smokers had no significant correlation with axial neck pain after cervical spine surgery. (ES = 1.54, 95% CI: 0.75–3.16, *p* = 0.236). After performing sensitivity analysis and removing the study by Liu et al.^44^ the only article on anterior cervical surgery, the heterogeneity was reduced to 38.9% (Fig. 8). Fixed-effects modeling revealed that the smoking group was significantly more associated with axial neck pain than the nonsmoking group (ES = 1.98, 95% CI: 1.25–3.12, *p* = 0.003).

#### Operation time

The operation time was provided in two studies^21,48^. No significant heterogeneity was observed, and a fixed-effects model was used (I^2^ = 0.0%, *p* = 0.955). Pooling of the results revealed no significant difference in operation time after cervical spine surgery between smokers and nonsmokers (WMD = 0.08, 95% CI: −5.54 to 5.71, *p* = 0.955; Supplementary Fig. S1a).

#### Estimated blood loss

The estimated blood loss was provided in three studies^2,11,48^. Significant heterogeneity was observed, and a random-effects model was used (I^2^ = 66.1%, *p* = 0.053). Pooling of the results revealed no significant difference in estimated blood loss after cervical spine surgery between smokers and nonsmokers (WMD = −5.31, 95% CI: −148.83 to 139.22, *p* = 0.943; Supplementary Fig. S1b). After performing leave-one-out sensitivity analysis, the heterogeneity did not change substantially and remained significant.

#### Length of hospital stay

The length of hospital stay was provided in four studies^2,11,21,48^. Significant heterogeneity was observed, and a random-effects model was used (I^2^ = 88.3%, *p* < 0.0001). Pooling of the results revealed no significant difference in the length of hospital stay after cervical spine surgery between smokers and nonsmokers (WMD = 1.01, 95% CI: −2.17 to 4.20, *p* = 0.534; Supplementary Fig. S1c) . After performing leave-one-out sensitivity analysis, the heterogeneity did not change substantially and remained significant.

#### VAS: neck pain

VAS-neck pain was reported in two studies^18,48^. No significant heterogeneity was observed, and a fixed-effects model was used (I^2^ = 0.0%, *p* = 0.530). Pooling of the results revealed no significant difference in VAS-neck pain after cervical spine surgery between smokers and non-smokers (WMD = −0.19, 95% CI: −1.19 to 0.81, *p* = 0.707; Supplementary Fig. S1d) .

#### VAS: arm pain

VAS-arm pain was reported in two studies^18,48^. No significant heterogeneity was observed, and a fixed-effects model was used (I^2^ = 0.0%, *p* = 1.000). Pooling of the results revealed no significant difference in VAS-arm pain after cervical spine surgery between smokers and nonsmokers (WMD = −0.50, 95% CI: −1.53 to 0.53, *p* = 0.343; Supplementary Fig. S1e).

#### NDI

NDI was reported in four studies^18,19,21,48^. Significant heterogeneity was observed, and a random-effects model was used (I^2^ = 96.4%, *p* < 0.0001). Pooling of the results revealed no significant difference in NDI after cervical spine surgery between smokers and nonsmokers (WMD = 11.46, 95% CI: −3.83 to 26.76, *p* = 0.142; Supplementary Fig. S1f.). After performing leave-one-out sensitivity analysis, the heterogeneity did not change substantially and remained significant.

#### JOA

JOA was reported in two studies^18,21^. Significant heterogeneity was observed, and a random-effects model was used (I^2^ = 89.4%, *p* = 0.002). Pooling of the results revealed no significant difference in JOA after cervical spine surgery between smokers and nonsmokers (WMD = −1.75, 95% CI: −5.27 to 1.78, *p* = 0.332; Supplementary Fig. S1g). Each specific result can be found in Table 4.

**Table 4:** Results of the meta-analysis.

Outcomes	No. of studies	ES/WMD	Meta-analyses		Heterogeneity		Model
			95%CIs	*p* value	I^2^ (%)	*p* value	Fixed
Overall complications	19	1.99	1.62–2.44	< 0.0001	45.3	0.017	Fixed
Respiratory complications	5	2.70	1.62–4.49	< 0.0001	31.2	0.213	Fixed
Reoperation	7	2.06	1.50–2.81	< 0.0001	41.4	0.115	Fixed
Fusion	16	0.97	0.94–1.00	0.097	38.2	0.061	Fixed
Dysphagia	8	1.49	1.06–2.10	0.022	46.9	0.068	Fixed
Wound infection	7	3.21	1.62–6.36	0.001	17.0	0.300	Fixed
Axial neck pain	2	1.98	1.25–3.12	0.003	38.9	0.201	Fixed
Operation time	2	0.08	−5.54–5.71	0.955	0.0	0.955	Fixed
Estimated blood loss	3	−5.31	−149.83–139.22	0.943	66.1	0.053	Random
Length of hospital stay	4	1.01	−2.17–4.20	0.534	88.3	< 0.0001	Random
VAS: neck pain	2	−0.19	−1.19–.0.81	0.707	0.0	0.530	Fixed
VAS: arm pain	2	−0.50	−1.53–0.53	0.343	0.0	1.000	Fixed
NDI	4	11.46	−3.83–26.76	0.142	96.4	< 0.0001	Random
JOA	2	−1.75	−5.27–1.78	0.332	89.4	0.002	Random

*Cis* confidence intervals, *ES* effect estimate, *WMD* weighted mean difference.

### Subgroup analysis

For primary outcomes, we conducted subgroup analysis based on the type of study. The results of four cohort studies were expressed as RR, and smoking had adverse effects on overall complications (RR = 2.55, 95% CI: 1.52–4.27, *p* < 0.0001). After removing one article, a total of 15 case–control studies were included. The results were expressed as ORs. Compared with nonsmokers, smokers were significantly more correlated with the overall complications after cervical spine surgery (OR = 1.90, 95% CI: 1.52–2.38, *p* < 0.0001).

### Publication bias

The Begg rank correlation test and Egger linear regression test indicated no evidence of significant publication bias among the included studies (Egger *p* = 0.266; Begg *p* = 0.266; Supplementary Fig. S2A, S2B).

## Discussion

The major purpose of the present meta-analysis was to determine whether smoking has adverse effects on surgical outcomes after cervical spine surgery. Our results suggest that smoking is associated with reoperation and postoperative complications, including dysphagia, axial neck pain, and wound infection. Compared with nonsmokers, smokers were more associated with overall postoperative complications and respiratory complications. There were no significant differences between smokers and nonsmokers concerning outcomes, including fusion, operation time, estimated blood loss, length of hospital stay, VAS-neck pain score, VAS-arm pain score, NDI score, or JOA score. Our results suggest that smoking might have adverse effects on surgical outcomes in patients who undergo cervical spine surgery.

Complications were the primary outcomes used to evaluate the safety of cervical spine surgery among smoking patients. Siemionow et al. conducted a study of 35 patients undergoing anterior and posterior cervical decompression and fusion and reported that smoking appeared to be the most critical factor related to perioperative complications; the risks for at least one perioperative complication were 50% and 31.6% for smokers and nonsmokers, respectively^7^. Lau et al. studied 160 patients undergoing anterior cervical corpectomy and found that smoking patients had longer hospital stays, more bleeding, a higher rate of pseudarthrosis, and more complications at 30 days than nonsmoking patients^11^. In contrast, Fehlings et al. analyzed data from the AOSpine North America Cervical Spondylotic Myelopathy Study and concluded that perioperative complications were not associated with smoking status^57^. Medvedev et al. reported the complication rates in smoking and nonsmoking patients of 23.5% and 39.8% (*p* < 0.0001), respectively^22^. Our pooled data showed that smoking was associated with increased postoperative complications, including dysphagia, airway obstruction, nerve palsy, reintubation, axial neck pain, wound infection, deep venous thrombosis and pneumonia.

We assessed perioperative outcomes, including fusion, operation time, estimated blood loss, and length of hospital stay in our meta-analysis and failed to find any significant difference between the smoking and nonsmoking groups. As measured by NDI, JOA, and VAS scores, functional recovery was similar between the two groups. This finding indicates that cervical spine surgery might offer similar functional outcomes in smoking patients. However, only two articles reported VAS-neck pain and JOA scores, one study found that smoking improved both VAS-neck pain and JOA scores, while the other found the opposite. Therefore, more articles can improve the accuracy of the conclusion, and the relatively small sample size limited the generalizability of this conclusion.

After cervical spine surgery, smokers were closely associated with reoperation. In this meta-analysis, given that functional improvement between the groups was similar, it is possible that reoperation was directly related to complications in smoking patients, including wound infection, respiratory complications, and pseudarthrosis. However, due to limited data, we did not perform a subgroup analysis based on the type of surgical procedure.

There are several potential explanations for the observed association between smoking and adverse effects on the surgical outcomes for patients after cervical spine surgery. First, cigarette smoke products have been shown to inhibit prostacyclin production, a potent vasodilator, and an inhibitor of platelet aggregation. This effect can lead to impaired blood flow and increased blood viscosity, which result in impaired blood supply^58–62^, and leads to decreased angiogenesis and epithelialization^63^. Moreover, inhibition of revascularization by nicotine was observed in a rabbit study, and this mechanism may retard cellular metabolism and promote tissue degeneration^64^.

Second, at the cellular level, nicotine has been shown to inhibit proliferation, differentiation, and collagen synthesis in osteoblasts^65^, which is the primary determinant of the tensile strength of a surgical wound^66^. Free radicals produced by burning cigarettes have been associated with cell membrane destabilization, impaired osteoblast mitochondrial oxidative function and local tissue hypoxia^58,67–71^.

Third, it is well-documented that smoking harms bone physiology, which result in decreased bone mineral density, impaired bone metabolism, and accelerated osteoporosis, with produces lower fusion rates^72^. Animal and in vitro studies found that nicotine impaired bone healing, retarded bone formation and growth, and decreased graft biomechanical properties^73,74^.

Finally, cigarette smoke contains many toxic ingredients. Nicotine, tar, and other components irritate mucous membranes of the respiratory tract and cause cilia of bronchial epithelial cells to become shorter and irregular, which can hinder the movement of ciliary bodies, reduce local resistance, and weaken phagocytosis and sterilization functions of alveolar phagocytes, which leads to bronchospasm and increased airway resistance^75^. For these reasons, smokers are susceptible to respiratory complications after cervical spine surgery. In addition, carbon monoxide combines with hemoglobin, which reduces the oxygen-carrying capacity of the blood, and hydrogen cyanide inhibits cytochrome c, and leads to inhibition of aerobic metabolism^76^.

To the best of our knowledge, our meta-analysis, on the basis of 16 cohort studies and 27 case–control studies, is the first, also the largest and most comprehensive assessment to investigate the association between smoking and outcomes of cervical spine surgery. The main strength of this systematic review and meta-analysis is the thorough literature search, careful study selection with strict inclusion criteria, and comprehensive assessment of methodological quality of included studies using the NOS, which is, currently, the accepted standard. In addition, we performed subgroup analysis according to the type of study for the primary outcomes. Although we found significant heterogeneity in several outcomes among the included studies, the sensitivity analysis showed no significant change, which suggested that the pooled estimate in our study was stable. Finally, publication bias was quantitatively evaluated using Begg’s and Egger’s linear regression tests.

This systematic review and meta-analysis have several limitations that are worthy of comment. First, studies included in our review spanned over two decades (1995 to 2021), during which advancements in cervical surgery techniques might have improved outcomes. Despite this, point estimates for earlier and more recent studies were similar. Second, all of the included studies were retrospective observational trials rather than randomized controlled trials. The inherent nature of observational trials may be associated with selective bias, which might have influenced our results. Third, in most studies, the definition of smoking was not standardized, and self-reporting introduces recall bias or response bias because nonsmokers may be current or former smokers. Therefore, the true impact of smoking may be larger than we have reported here. Moreover, the definition of complications was not uniform and might introduce an additional source of bias. Fourth, since most of the information collected was not used to answer specific questions, all characteristics of smoker and nonsmoker cohorts such as age, sex, BMI, ethnic group, indications for surgery, and comorbidities, were not necessarily consistently matched, leaving some possible residual confusion, resulting in high heterogeneity. Moreover, due to the limited number of articles, we did not compare the various types of cervical spine surgeries in detail. In addition, only two studies reported operation time, VAS-neck pain, VAS-arm pain and JOA, and only three studies reported estimated blood loss and axial neck pain. Their results were based on a very small number of studies, which may lack reference value. Finally, we do not know how investigators confirmed that their patients did not smoke before or after surgery or even if they quit smoking before surgery, which may have impacted the evaluated results.

One study analyzed the pack-year history and found that, after lumbar surgery, nicotine exposure was associated with an increased risk of disease, and there was a dose–response trend; however, this trend was not significant^77^. In contrary, another study did not support this view and found that after anterior cervical discectomy and fusion, pack-years were not significantly associated with greater odds of developing any one complication or any major complication^78^. This may be related to differences in the number, characteristics, surgical sites, and follow-up time of the population included in the study. Therefore, there is an urgent need for further high-quality studies that are sufficiently prepared and designed with sufficient detail to adjust for multiple confounders and allow exploration of dose–response relationships.

Some researchers reported that preoperative smoking cessation might improve surgery outcomes and could lower medical costs by decreasing postoperative complications and length of post surgical hospital stay among smokers^11,79^. Sørensen et al. performed a meta-analysis and found that smoking cessation reduced the risk of surgical site infection in plastic and general surgery patients by more than half^80^. Andersen et al. found that quitting smoking significantly increased the rate of fusion after spinal surgery compared to those who continued to smoke, bringing it close to the level of nonsmokers^81^. This may be related to the rapid recovery of local tissue oxygenation and metabolism after smoking cessation^82^. Therefore, it is theoretically necessary to quit smoking before elective surgery.

Nevertheless, the optimal timing to quit smoking remains a matter of considerable debate. A study showed that quitting smoking 1 to 2 months before surgery can significantly reduce the perioperative risk^77^. Some studies indicated that smoking cessation must be at least 4 weeks before surgery to be effective^83,84^. Another study said that smoking cessation should be carried out at least 2 months before coronary artery bypass to maximize the reduction of postoperative respiratory complications^85^. Jung et al. found that preoperative smoking cessation for at least 2 weeks will help to reduce the incidence of postoperative complications in gastric cancer surgery^86^. Thus, exploring the optimal timing to quit smoking before the operation should determine future efforts.

## Conclusions

Compared with nonsmokers, smokers seem to be more significantly associated with overall complications, respiratory complications, reoperation, longer hospital stay, dysphagia, wound infection and axial neck pain after cervical spine surgery. Our results suggest that smoking is closely related to adverse consequences after cervical spine surgeries. It is crucial to provide timely smoking cessation advice and explanation to patients before elective cervical surgery.

## Supplementary Information

Supplementary Information 1.

Supplementary Information 2.

Supplementary Information 3.

Supplementary Information 4.

Supplementary Information 5.

Supplementary Information 6.

Supplementary Information 7.

Supplementary Information 8.

Supplementary Information 9.

Supplementary Table 1.

Supplementary Table 2.

Supplementary Legends.

## Data Availability

All data generated or analyzed during this study are included in this published article and its supplementary information files.

## Abbreviations

CIs: Confidence intervals
ES: Effect estimate
OR: Odds ratio
RR: Relative risk
NR: Not reported
NOS: Newcastle–Ottawa Scale
ACDF: Anterior cervical discectomy and fusion
SD: Standard Deviation
NDI: Neck Disability Index
JOA: Japanese Orthopedic Association Scores
VAS: Visual Analog Scale
EBL: Estimated blood loss
CSF: Cerebrospinal fluid
DVT: Deep venous thrombosis
