# Supplementary figures and images for: Relationship between smoking and postoperative complications of cervical spine surgery: a systematic review and meta-analysis
Source: Sci Rep. 2022 Jun 2;12:9172. doi: 10.1038/s41598-022-13198-x (PMC9163175; doi:10.1038/s41598-022-13198-x)

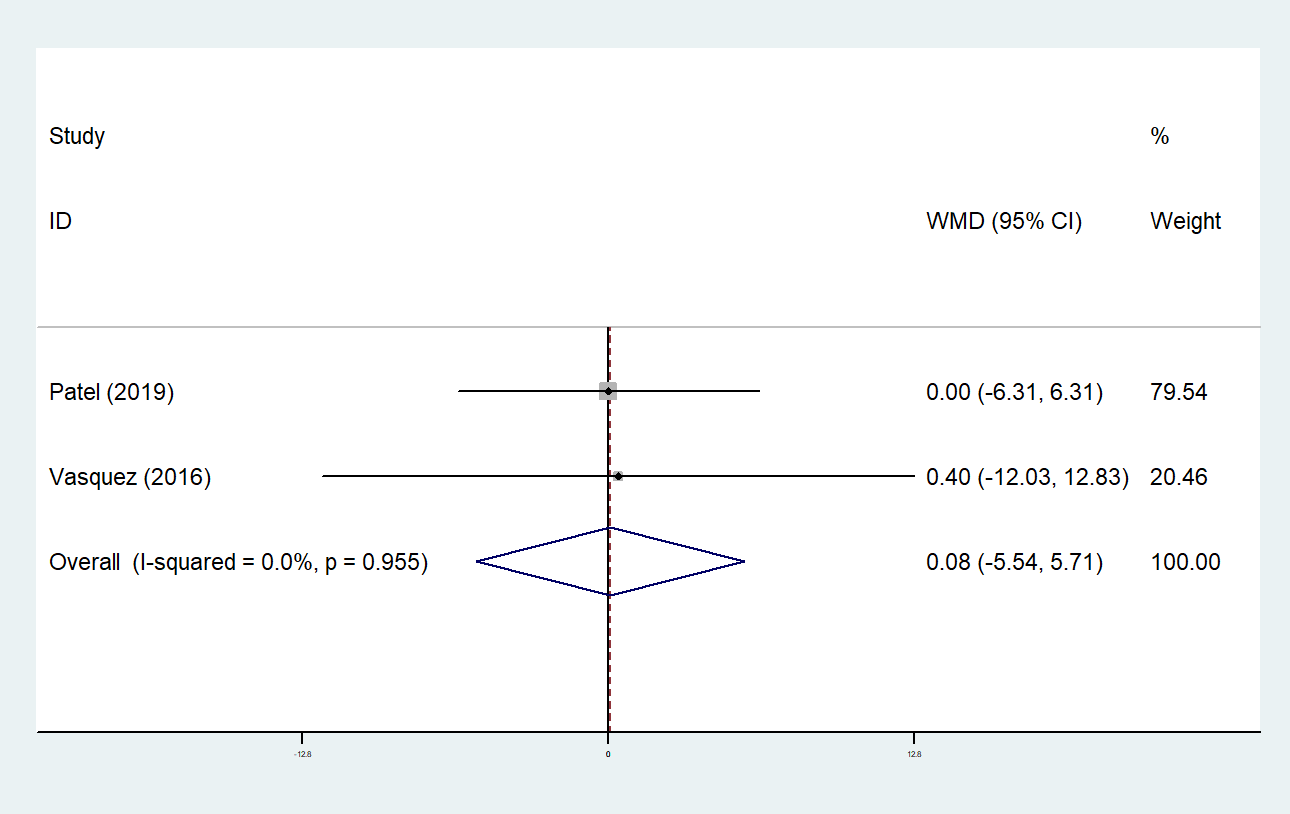

Supplement: Supplementary file 1 — Supplementary Information 1. [file 41598_2022_13198_MOESM1_ESM.tif]

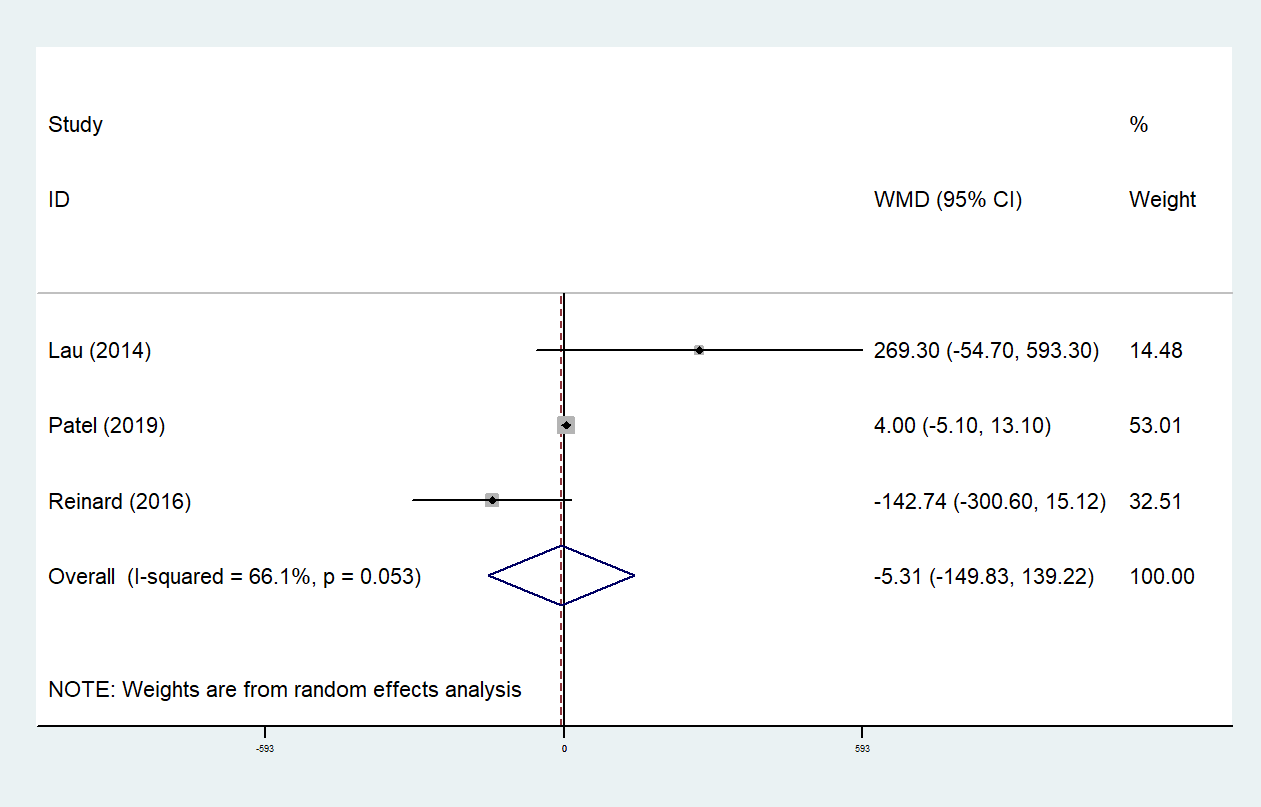

Supplement: Supplementary file 2 — Supplementary Information 2. [file 41598_2022_13198_MOESM2_ESM.tif]

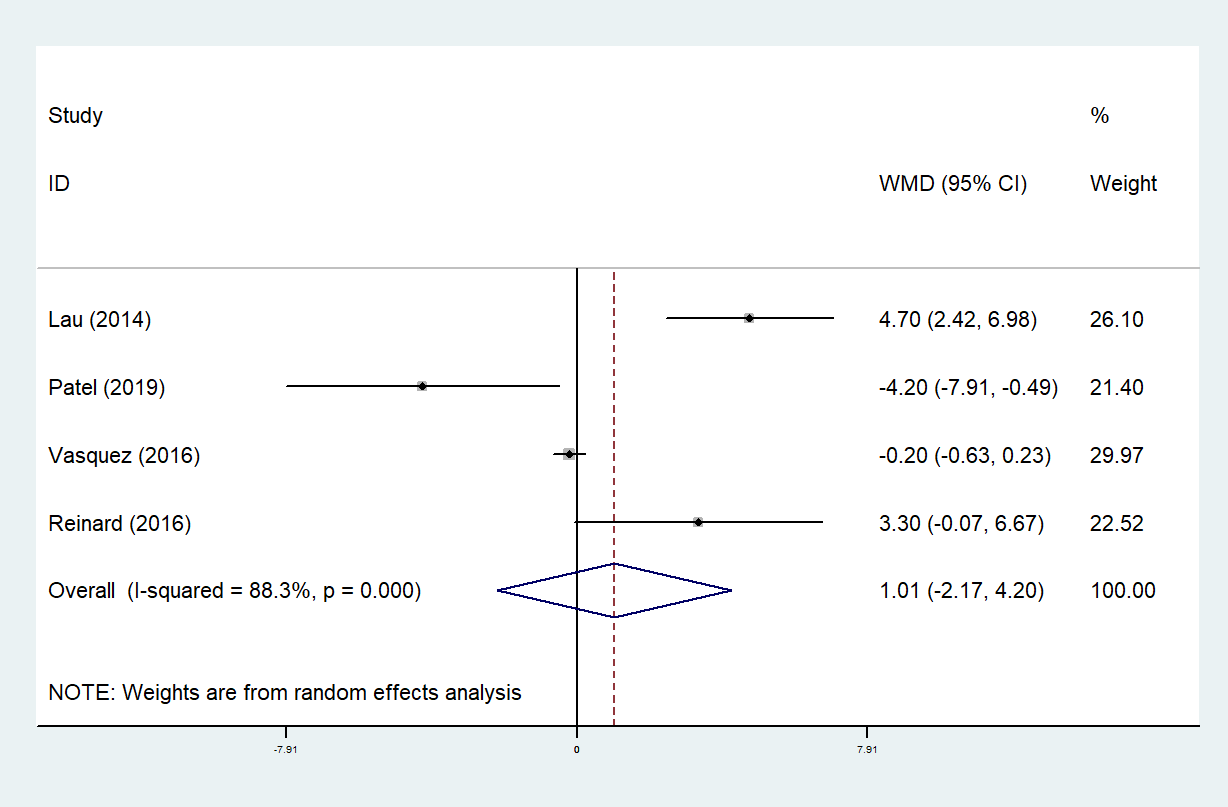

Supplement: Supplementary file 3 — Supplementary Information 3. [file 41598_2022_13198_MOESM3_ESM.tif]

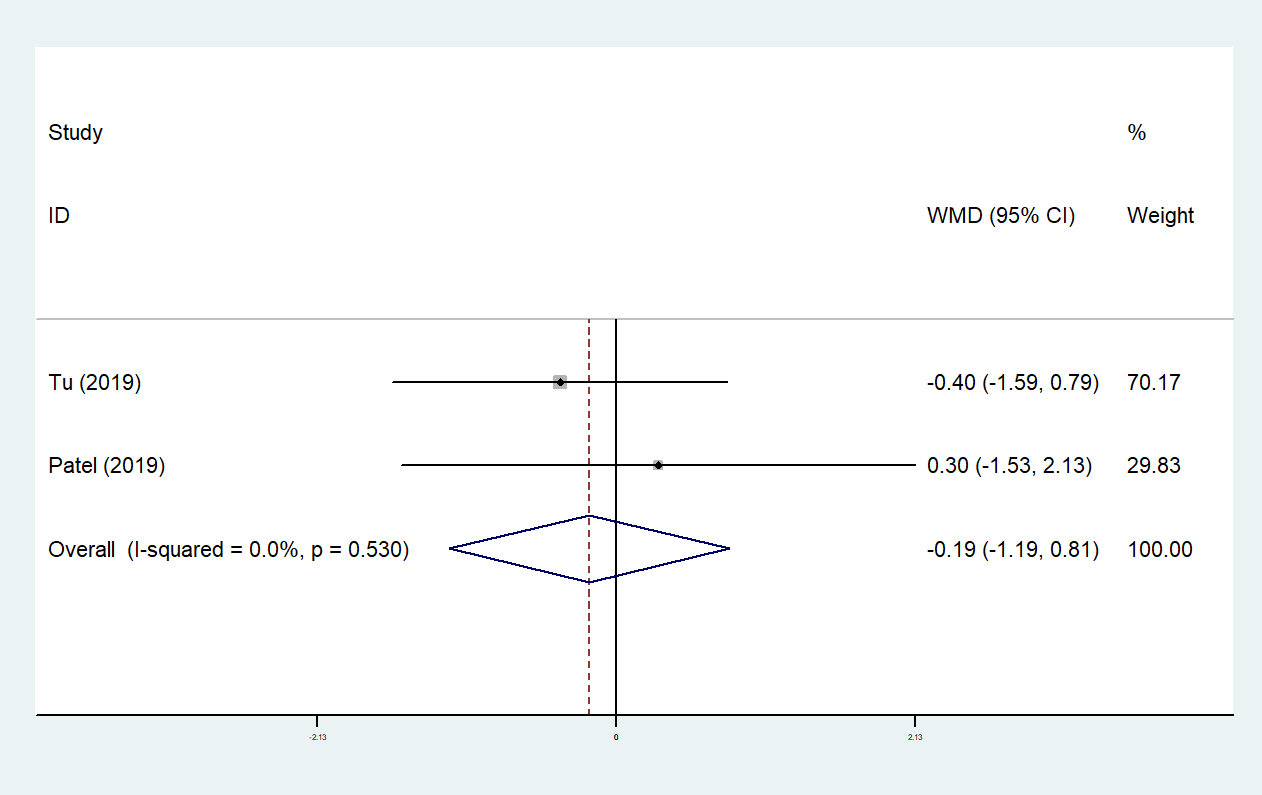

Supplement: Supplementary file 4 — Supplementary Information 4. [file 41598_2022_13198_MOESM4_ESM.tif]

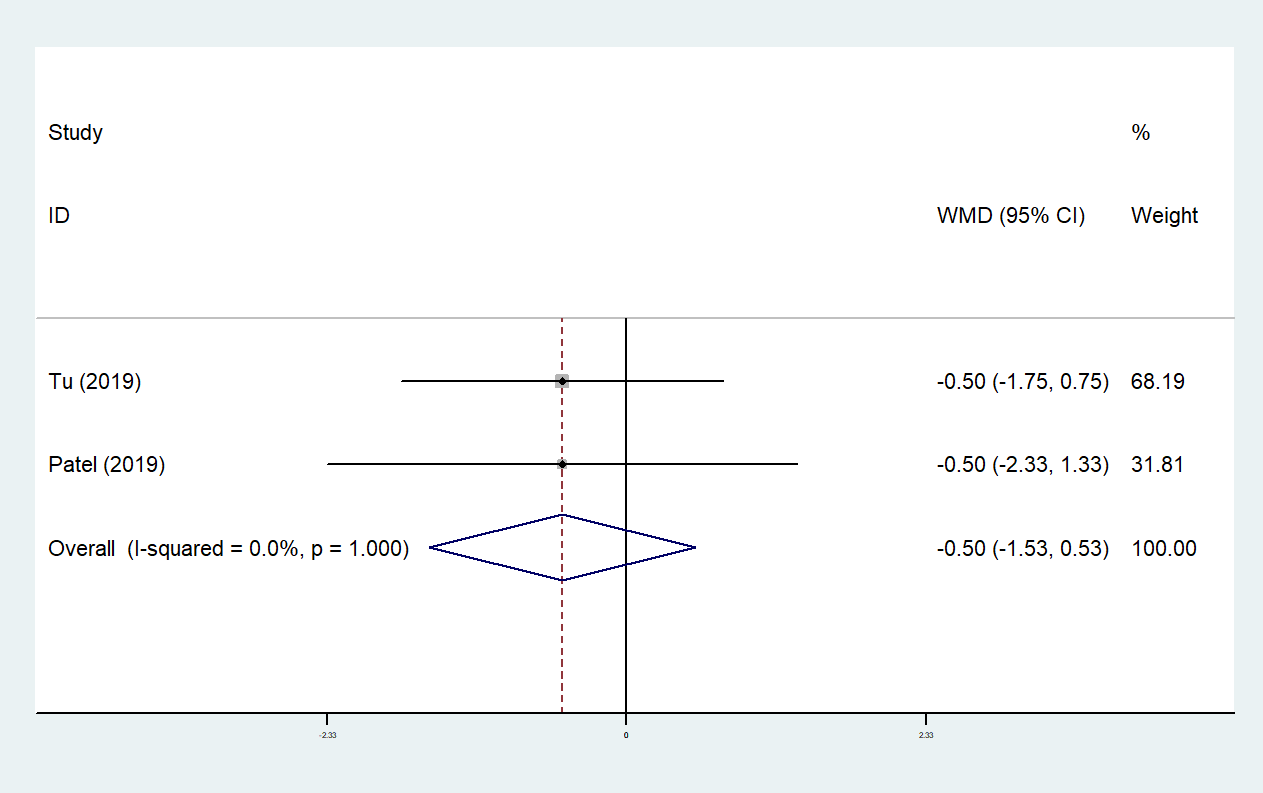

Supplement: Supplementary file 5 — Supplementary Information 5. [file 41598_2022_13198_MOESM5_ESM.tif]

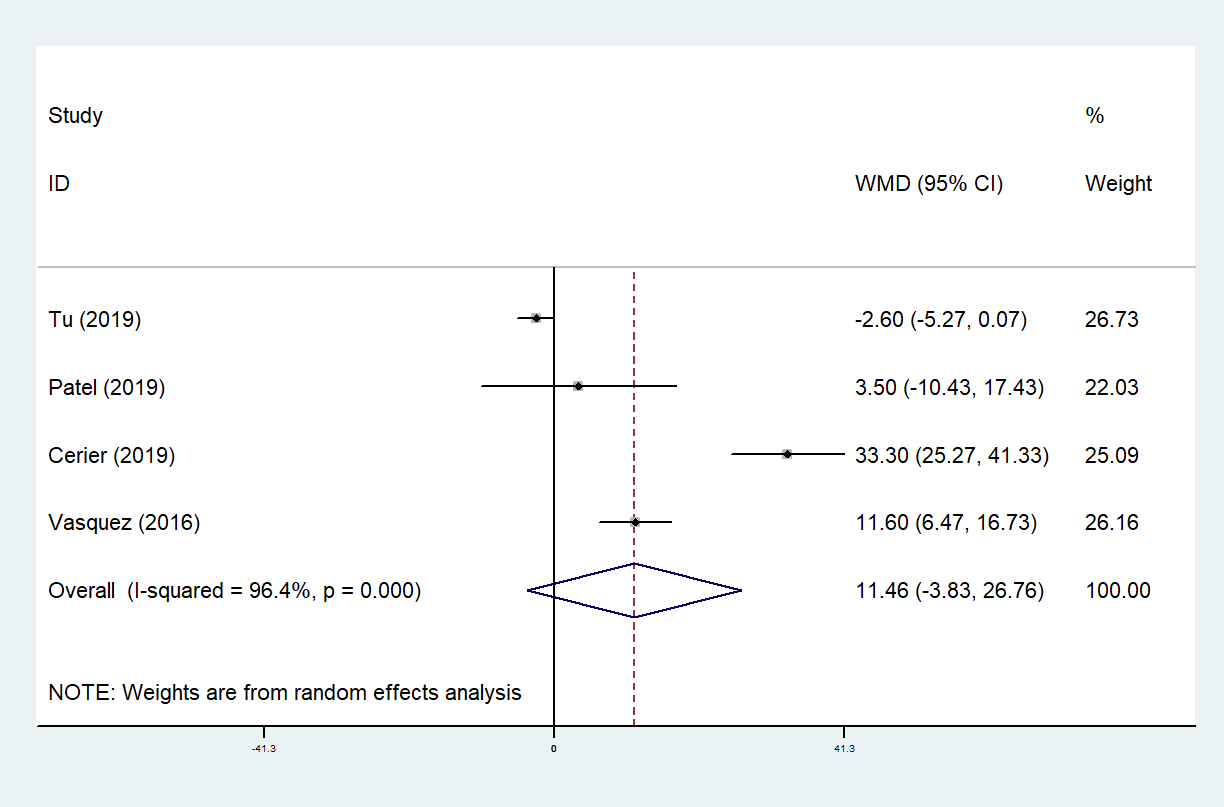

Supplement: Supplementary file 6 — Supplementary Information 6. [file 41598_2022_13198_MOESM6_ESM.tif]

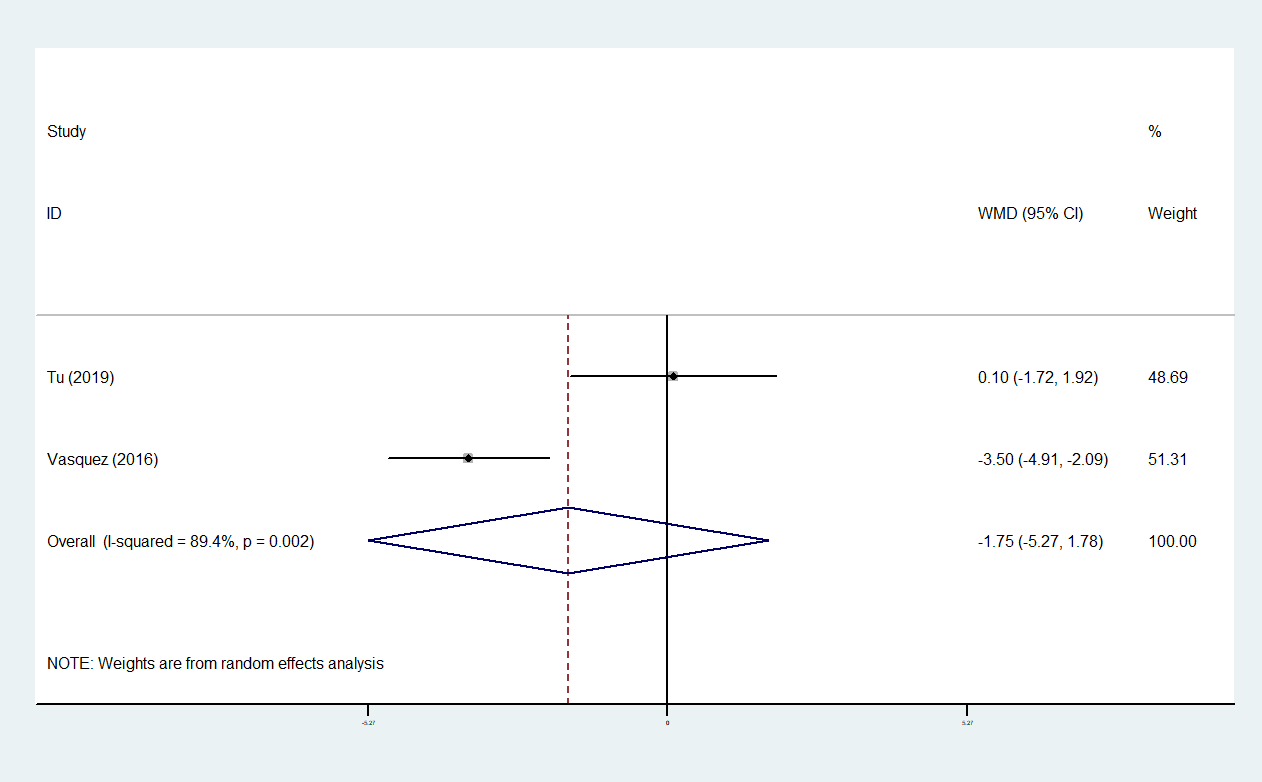

Supplement: Supplementary file 7 — Supplementary Information 7. [file 41598_2022_13198_MOESM7_ESM.tif]

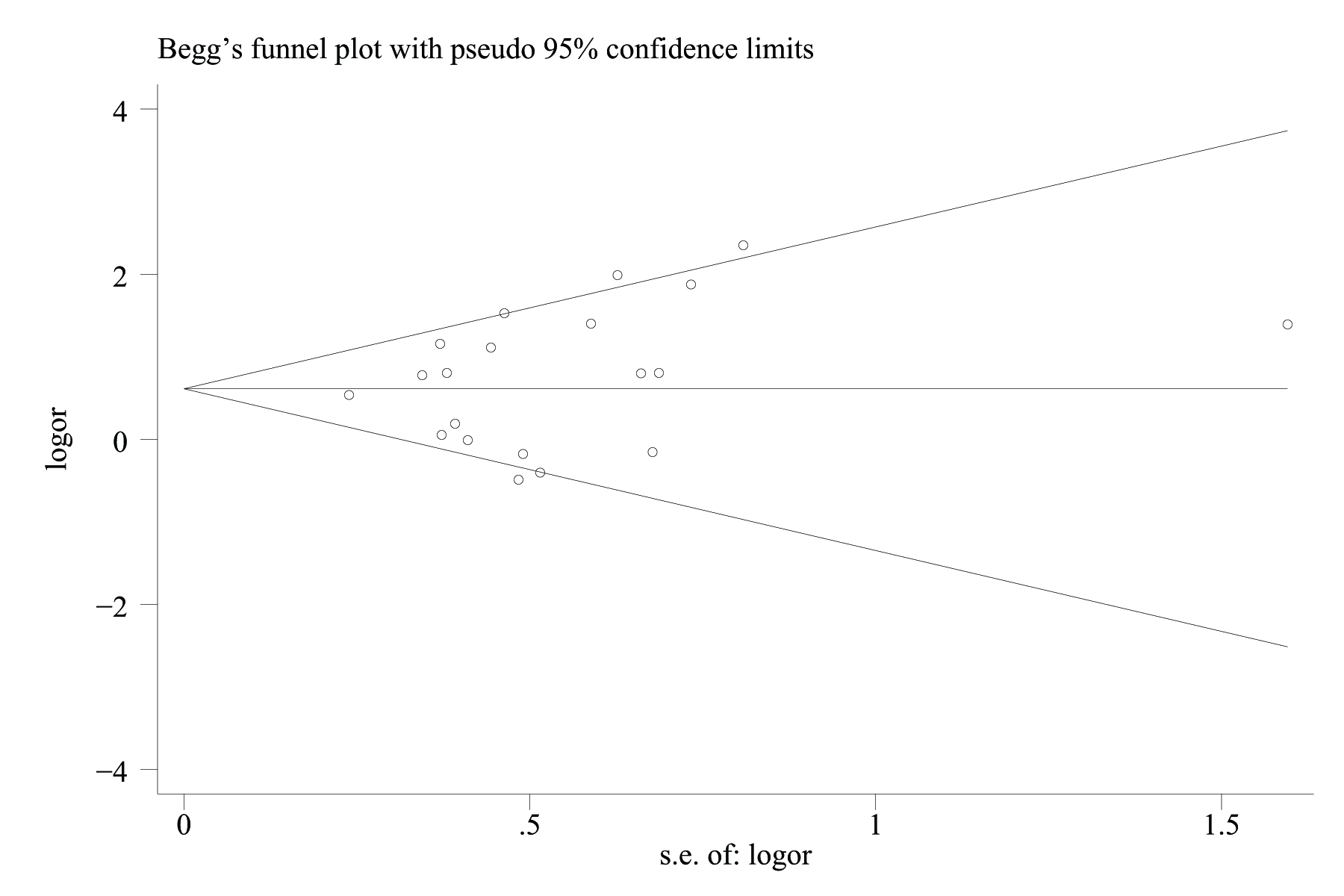

Supplement: Supplementary file 8 — Supplementary Information 8. [file 41598_2022_13198_MOESM8_ESM.tif]

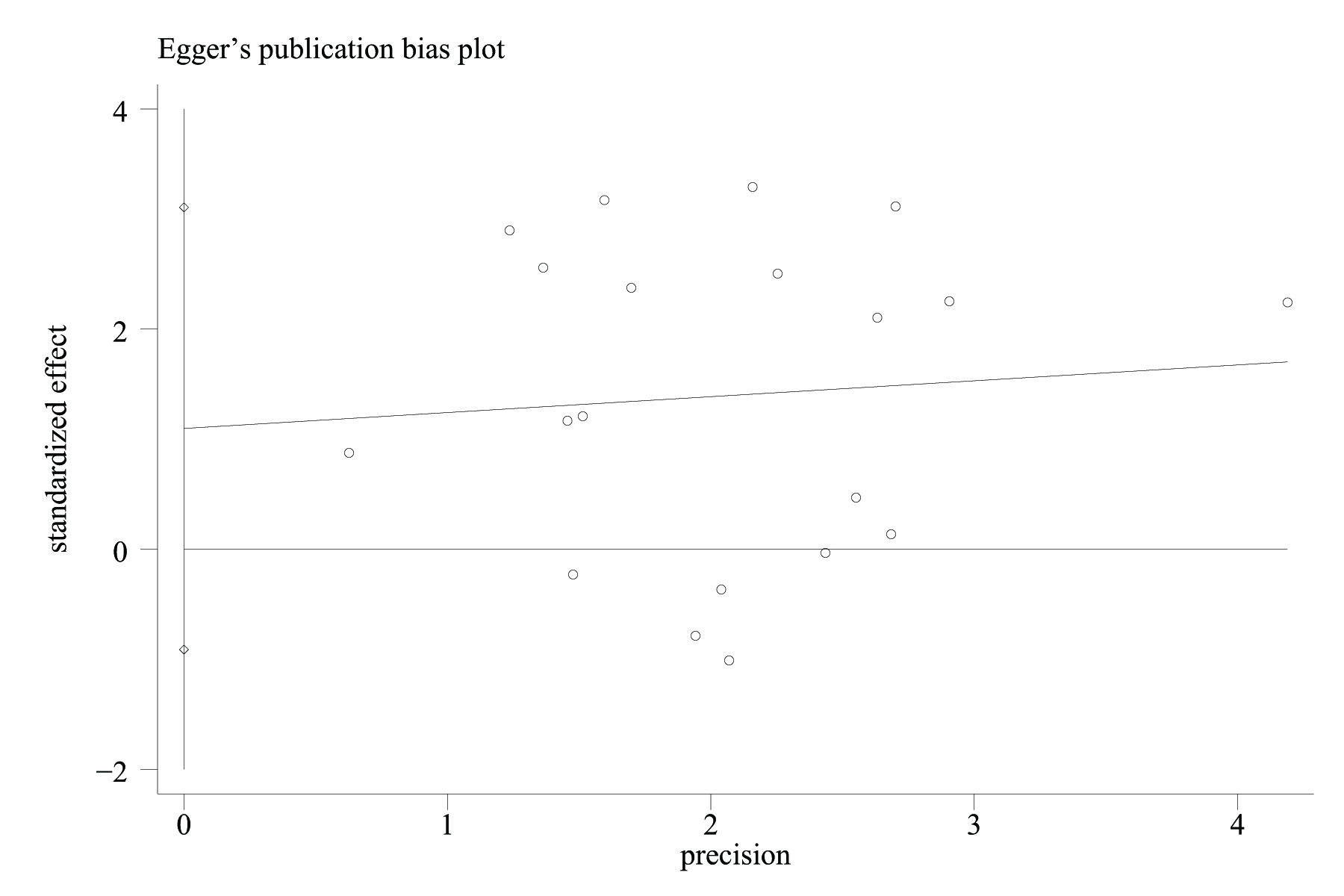

Supplement: Supplementary file 9 — Supplementary Information 9. [file 41598_2022_13198_MOESM9_ESM.tif]
