# Supplementary material for: Relationship between smoking and postoperative complications of cervical spine surgery: a systematic review and meta-analysis
Source: Sci Rep. 2022 Jun 2;12:9172. doi: 10.1038/s41598-022-13198-x (PMC9163175; doi:10.1038/s41598-022-13198-x)
Supplement: Supplementary file 10 — Supplementary Table 1. [file 41598_2022_13198_MOESM10_ESM.docx]

Supplementary table 1. Dichiotomous variable raw data

| Outcomes | author | year | number of smokers | | number of nonsmokers | |
| --- | --- | --- | --- | --- | --- | --- |
|  |  |  | event | noevent | event | noevent |
| Overall Complications | Bose | 2001 | 4 | 42 | 6 | 54 |
|  | Eubanks | 2011 | 6 | 35 | 3 | 114 |
|  | Lau | 2014 | 25 | 56 | 7 | 72 |
|  | Tu | 2019 | 4 | 16 | 9 | 80 |
|  | Badiee | 2021 | 5 | 22 | 7 | 225 |
|  | Chen | 2015 | 12 | 56 | 32 | 157 |
|  | Dube | 2018 | 24 | 20 | 58 | 105 |
|  | Huang | 2020 | 20 | 31 | 22 | 108 |
|  | Kang | 2014 | 11 | 30 | 11 | 20 |
|  | Klement | 2016 | 2 | 0 | 15 | 12 |
|  | Lee | 2015 | 34 | 299 | 44 | 661 |
|  | Liang | 2017 | 15 | 44 | 21 | 137 |
|  | Nakashima | 2013 | 14 | 41 | 11 | 98 |
|  | Pahys | 2013 | 7 | 119 | 2 | 355 |
|  | Reinard | 2016 | 15 | 32 | 13 | 17 |
|  | Riederman | 2017 | 10 | 26 | 46 | 118 |
|  | Sagi | 2002 | 7 | 120 | 12 | 172 |
|  | Schnee | 1997 | 4 | 62 | 5 | 173 |
|  | Siemionow | 2014 | 8 | 8 | 6 | 13 |
|  | Wang | 2017 | 25 | 21 | 5 | 17 |
| Respiratory Complications | Bose | 2001 | 0 | 46 | 1 | 59 |
|  | Lau | 2014 | 10 | 71 | 0 | 79 |
|  | Dube | 2018 | 24 | 20 | 58 | 105 |
|  | Nakashima | 2013 | 14 | 41 | 11 | 98 |
|  | Sagi | 2002 | 7 | 120 | 12 | 172 |
|  | Siemionow | 2014 | 6 | 10 | 0 | 19 |
| Fusion | An | 1995 | 18 | 16 | 31 | 12 |
|  | Bose | 2001 | 45 | 1 | 58 | 2 |
|  | Cerier | 2019 | 14 | 9 | 21 | 17 |
|  | Goldberg | 2002 | 15 | 15 | 35 | 15 |
|  | Hilibrand | 2001 | 34 | 21 | 110 | 25 |
|  | Lau | 2014 | 55 | 7 | 67 | 3 |
|  | Luszczyk | 2013 | 142 | 14 | 382 | 35 |
|  | Mangan | 2021 | 56 | 7 | 73 | 14 |
|  | Martin | 1999 | 62 | 13 | 193 | 21 |
|  | Suchomel | 2004 | 44 | 4 | 30 | 1 |
|  | Wang | 1999 | 11 | 1 | 64 | 4 |
|  | Agrillo | 2002 | 13 | 6 | 23 | 3 |
|  | Bergin | 2021 | 41 | 7 | 242 | 36 |
|  | Groff | 2003 | 50 | 5 | 88 | 1 |
|  | Ren | 2020 | 100 | 6 | 177 | 12 |
|  | Wang | 2000 | 4 | 2 | 47 | 5 |
| Dysphagia | Bose | 2001 | 1 | 45 | 2 | 58 |
|  | Tu | 2019 | 1 | 19 | 4 | 85 |
|  | Chen | 2015 | 12 | 56 | 32 | 157 |
|  | Huang | 2020 | 20 | 31 | 22 | 108 |
|  | Kang | 2014 | 11 | 27 | 11 | 53 |
|  | Reinard | 2016 | 15 | 32 | 13 | 17 |
|  | Riederman | 2017 | 9 | 27 | 42 | 122 |
|  | Wang | 2017 | 25 | 21 | 5 | 17 |
| Wound Infection | Tu | 2019 | 1 | 19 | 2 | 87 |
|  | Eubanks | 2011 | 2 | 39 | 0 | 117 |
|  | Lau | 2014 | 4 | 77 | 2 | 77 |
|  | Pahys | 2013 | 7 | 118 | 2 | 356 |
|  | Schnee | 1997 | 4 | 62 | 5 | 73 |
|  | Siemionow | 2014 | 1 | 15 | 0 | 19 |
|  | Badiee | 2021 | 3 | 24 | 5 | 227 |
| Axial Neck Pain | Kimura | 2018 | 20 | 19 | 31 | 86 |
|  | Liu | 2019 | 9 | 30 | 15 | 34 |
|  | Zhang | 2020 | 29 | 39 | 58 | 123 |
