# Supplementary material for: Relationship between smoking and postoperative complications of cervical spine surgery: a systematic review and meta-analysis
Source: Sci Rep. 2022 Jun 2;12:9172. doi: 10.1038/s41598-022-13198-x (PMC9163175; doi:10.1038/s41598-022-13198-x)
Supplement: Supplementary file 11 — Supplementary Table 2. [file 41598_2022_13198_MOESM11_ESM.docx]

Supplementary table 2. Continuous variable raw data

| Outcomes | author | year | smoking | | | nonsmoking | | |
| --- | --- | --- | --- | --- | --- | --- | --- | --- |
|  |  |  | mean | SD | n | mean | SD | n |
| Operation Time (min) | Patel | 2019 | 51.5 | 14.8 | 25 | 51.5 | 16.3 | 167 |
|  | Vasquez | 2016 | 162 | 60 | 123 | 161.6 | 62 | 350 |
| Estimated Blood Loss (mL) | Lau | 2014 | 518.9 | 1035.8 | 40 | 249.6 | 199.6 | 79 |
|  | Patel | 2019 | 34 | 22.7 | 25 | 30 | 12.7 | 167 |
|  | Reinard | 2016 | 340.43 | 339.88 | 47 | 483.17 | 347.68 | 30 |
| Length of Hospital Stay (h) | Lau | 2014 | 9.5 | 6.9 | 40 | 4.8 | 3.6 | 79 |
|  | Patel | 2019 | 9.6 | 8.1 | 25 | 13.8 | 12.6 | 167 |
|  | Vasquez | 2016 | 1.9 | 2.1 | 123 | 2.1 | 2.1 | 350 |
|  | Reinard | 2016 | 12.1 | 9.3 | 47 | 8.8 | 5.8 | 30 |
| VAS-neck pain score | Tu | 2019 | 2.2 | 2.4 | 20 | 2.6 | 2.7 | 89 |
|  | Patel | 2019 | 3.2 | 4.4 | 25 | 2.9 | 4 | 167 |
| VAS-arm pain score | Tu | 2019 | 1.6 | 2.6 | 20 | 2.1 | 2.5 | 89 |
|  | Patel | 2019 | 3.2 | 4.4 | 25 | 3.7 | 4.1 | 167 |
| Neck Disability Index score | Tu | 2019 | 5.2 | 5.1 | 20 | 7.8 | 7 | 89 |
|  | Patel | 2019 | 26.1 | 34 | 25 | 22.6 | 26.7 | 167 |
|  | Cerier | 2019 | 51.1 | 14.6 | 23 | 17.8 | 16.9 | 38 |
|  | Vasquez | 2016 | 33.9 | 24.5 | 123 | 22.3 | 26.2 | 350 |
| Japanese Orthopedic Association Scores | Tu | 2019 | 14.8 | 4 | 20 | 14.7 | 2.3 | 89 |
|  | Vasquez | 2016 | 13.7 | 6.9 | 123 | 17.2 | 6.8 | 350 |

SD=standard deviation
