# Supplementary material for: Relationship between smoking and postoperative complications of cervical spine surgery: a systematic review and meta-analysis
Source: Sci Rep. 2022 Jun 2;12:9172. doi: 10.1038/s41598-022-13198-x (PMC9163175; doi:10.1038/s41598-022-13198-x)
Supplement: Supplementary file 12 — Supplementary Legends. [file 41598_2022_13198_MOESM12_ESM.docx]

**Supplementary Fig.1a** Forest plot showing the effect of smoking on operation time. WMD, weighted mean difference; CI, confidence interval.

**Supplementary Fig.1b** Forest plot showing the effect of smoking on estimated blood loss. WMD, weighted mean difference; CI, confidence interval.

**Supplementary Fig.1c** Forest plot showing the effect of smoking on length of hospital stay. WMD, weighted mean difference; CI, confidence interval.

**Supplementary Fig.1d** Forest plot showing the effect of smoking on Visual Analog Scale-neck pain score. WMD, weighted mean difference; CI, confidence interval.

**Supplementary Fig.1e** Forest plot showing the effect of smoking on Visual Analog Scale-arm pain score. WMD, weighted mean difference; CI, confidence interval.

**Supplementary Fig.1f** Forest plot showing the effect of smoking on Neck Disability Index score. WMD, weighted mean difference; CI, confidence interval.

**Supplementary Fig.1g** Forest plot showing the effect of smoking on Japanese Orthopedic Association Scores. WMD, weighted mean difference; CI, confidence interval.

**Supplementary Fig.2a** Begg’s funnel plot to evaluate the publication bias of overall complications.

**Supplementary Fig.2b** Egger’s publication bias plot to evaluate the publication bias of overall complications.
